# Increased or decreased numbers of CpG dinucleotide motifs in the genome of influenza A virus do not affect *in vitro* virus phenotype

**DOI:** 10.1128/jvi.00047-26

**Published:** 2026-06-22

**Authors:** Balasubramanian Susma, Monique Spronken, Stefan van Nieuwkoop, Bernike Kalverda, Alexander P. Gultyaev, Ron A. M. Fouchier, Bernadette G. van den Hoogen

**Affiliations:** 1 Department of Viroscience, Erasmus Medical Center 6993, Rotterdam, the Netherlands; University of Freiburg, Freiburg, Germany

**Keywords:** influenza virus, CpG motifs, evolutionary pressure

## Abstract

Cytosine–phosphate–guanine (CpG) dinucleotide motifs are the most underrepresented motifs in RNA virus genomes, including those of the influenza A viruses (IAV). Previous studies have indicated that increasing the number of CpG motifs in IAV genomes negatively impacts viral replication. However, outcomes of these studies have been inconsistent, most likely due to variations in experimental designs. Here, the impact of altered numbers of CpG motifs in the IAV genome was investigated by carefully designing mutants with either increased or decreased frequencies of CpG motifs. Importantly, CpG mutations were excluded from regions essential for viral replication, such as predicted RNA secondary structures and alternative open reading frames, and only mutations naturally occurring in viral isolates were introduced. The resulting mutant viruses showed no significant differences in replication efficiency in human respiratory epithelial cells, nor in their ability to activate the innate immune response. Furthermore, after repeated passage in both human respiratory epithelial (A549) and MDCK cells, the introduced mutations remained stable, indicating no selection pressure on CpG motifs *in vitro*. This is the first study to assess the effects of genome-wide increases and decreases in CpG content in IAV while preserving essential genomic structures. Under these controlled conditions, altering CpG motif frequency did not alter the viral phenotype.

**IMPORTANCE:**

CpG motifs are underrepresented in RNA virus genomes, including those of influenza A virus (IAV). Previous work suggested that increasing CpG content attenuates IAV and could be exploited to design live-attenuated vaccines. However, previous work focused on single IAV genes, did not control for secondary effects, nor included mutants with decreased CpG frequencies. Here, we engineered mutants with decreased or increased CpG frequencies across multiple viral genes. Only naturally occurring mutations were introduced, while regions involved in RNA secondary structures or alternative open reading frames were preserved. Altered CpG frequencies had no significant effect on IAV replication and did not alter innate immune responses. Repeated viral passaging in immune-competent cells revealed the absence of natural selection on CpG motifs. These results demonstrate that altering CpG content does not affect IAV phenotype and challenge the rationale of using CpG enrichment to design live-attenuated IAV vaccines.

## INTRODUCTION

Influenza A virus (IAV) is a highly infectious respiratory pathogen, responsible for an estimated 3–5 million cases of severe disease and 290,000–650,000 deaths annually (1, 2). To combat IAV infections, it is crucial to understand their replication, pathogenesis, and evolution. IAV belongs to the family of *Orthomyxoviridae* and contains a genome composed of eight segments of single-stranded, negative-sense RNA. These segments encode more than 10 viral proteins, including RNA-dependent RNA polymerase proteins (PB2, PB1, and PA), nucleoprotein (NP), surface glycoproteins hemagglutinin (HA) and neuraminidase (NA), matrix protein 1 (M1), matrix protein 2 (M2), non-structural protein 1 (NS1), and nuclear export protein (NEP or NS2), and strain-dependent small secondary open reading frames (3, 4).

The genomes of vertebrate organisms are characterized by biased nucleotide compositions, with cytosine–phosphate–guanine (CpG)-dinucleotide motifs significantly underrepresented, occurring at only 20%–25% of the expected frequency (5). This is mainly due to methylation of cytosines and spontaneous deamination of 5-methylcytosine to thymine, resulting in C → T mutations. CpG underrepresentation is rare in invertebrates, bacteria, and fungi in which CpG methylation is uncommon. Unmethylated CpG motifs play a crucial role in activating the vertebrate immune system by helping to discriminate self from non-self-DNA (6). Viruses often evade host innate immune detection by mimicking the nucleotide composition of their host genome (7). Evolutionary pressure exerted by vertebrate host immune systems is also thought to play a significant role in driving the underrepresentation of CpG motifs in genomes of many RNA viruses (8, 9), including IAV (8, 10, 11), and is found to be independent of their genomic organization (dsRNA or ssRNA, positive or negative sense) and site of replication (cytoplasm or nucleus) (7). In 2008, Greenbaum et al. described changes in dinucleotide motifs in the IAV genome during virus evolution in humans since 1918 (8). This study revealed that CpG motifs were the most underrepresented among the 16 possible dinucleotide motifs in the H1N1 IAV genome, with the frequency decreasing over time following the introduction of this virus from avian hosts to humans (8). In contrast, the frequency of CpG motifs in influenza B virus genomes remained very low throughout recent evolution. It was hypothesized that after introduction into the human host centuries ago, influenza B viruses fully adapted to humans, resulting in a reduction of CpG motifs to approximately half the number of those in IAV (8, 12). Initially, CpG underrepresentation was thought to be the result of species-specific adaptation, but a study by Gu et al. revealed that CpG motifs were highly underrepresented in both human and avian influenza viruses (13). This observation suggested that a general selection pressure acts on CpG motifs in both human and avian hosts, possibly due to CpG motifs being recognized as non-self by the immune systems of both host species.

The effect of the number of CpG motifs on virus phenotype has also been studied for various other viruses, including echovirus 7, poliovirus, human immunodeficiency virus (HIV), and Zika virus. These studies consistently demonstrated that artificially increasing the number of CpG motifs in virus genomes led to virus attenuation (14–17).

Zinc-finger antiviral protein (ZAP) has been proposed as a cellular sensor that targets viral CpG dinucleotides in RNAs, and CpG-dependent attenuation has been associated with ZAP activity in several studies (18, 19). However, ZAP-induced attenuation has not consistently correlated with the presence of a high number of CpG motifs in the HIV genome (20) or the influenza virus genome (21). Several aspects of ZAP’s mechanism of action, therefore, remain incompletely understood, including the precise contribution of CpG dinucleotide frequency to antiviral activity. In most published studies, the design of the CpG mutants involved introducing as many CpG motifs as possible without disturbing the coding potential of the gene (22, 23). However, the opposite approach of reducing the number of CpG motifs to test for a potential beneficial effect on virus replication was only done in echovirus studies (14). A recent study revealed that attenuation of an IAV with enriched CpG content resulted from compensatory substitutions introduced to preserve mononucleotide frequency, rather than from CpG enrichment itself, demonstrating the importance of improved experimental design involving both gain-of-function and loss-of-function approaches when creating and testing CpG mutant viruses (21).

The present study aimed to assess the impact of altered CpG motif frequencies by comparing viruses engineered with either increased or decreased CpG numbers, introducing mutations across six gene segments of the IAV genome. The M and NS genes, along with other regions constrained by RNA secondary structure, were left unaltered. To minimize potential functional impact on virus replication unrelated to the genome bias itself, CpG motifs were introduced only at positions naturally observed to be variable in IAVs circulating since 1900. This approach resulted in two mutant variants: one with high CpG content (“CpG-high,” with 531 CpG motifs) and one with low CpG content (“CpG-low,” with 148 CpG motifs) compared to the parental wild-type virus (273 CpG motifs). These mutants were evaluated for replication kinetics and activation of the innate immune response in mammalian A549 cells.

## RESULTS

### Strategy for generating IAV mutants with modified numbers of CpG motifs

**Fig 1:**
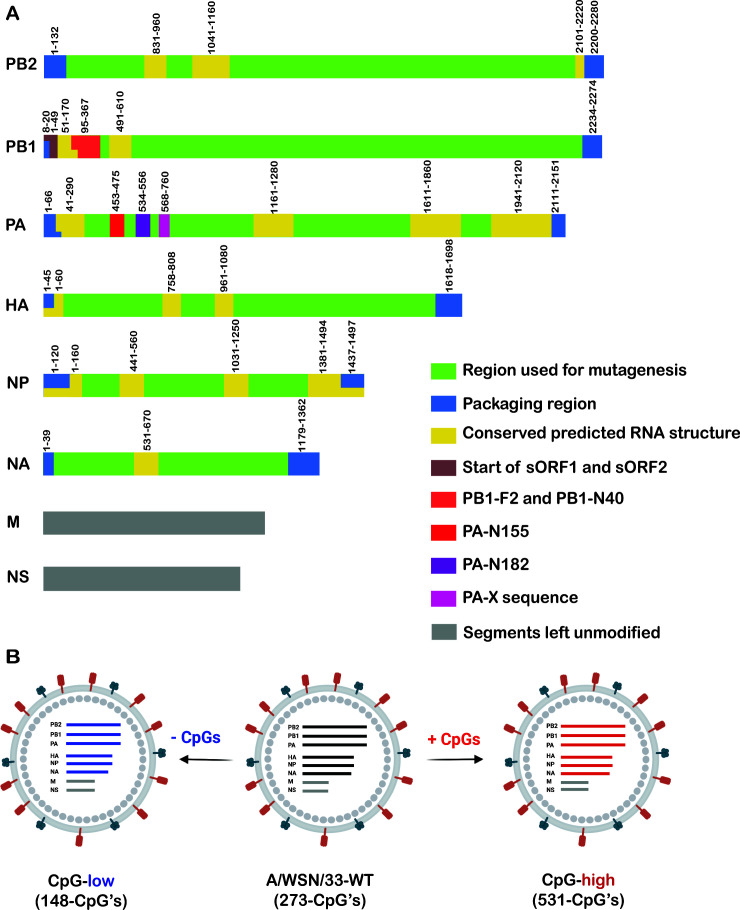
(**A**) Schematic representation of the strategy for generating IAV mutants with altered numbers of CpG motifs. Synonymous mutations were introduced to change the number of CpG motifs only in the green regions of the gene segments. Yellow regions: predicted secondary RNA structures; blue regions: involved in RNA packaging into the virion; purple/orange/red/violet regions: alternate ORFs of accessory proteins present in PB1 and PA segments. M and NS (gray) were excluded as they contain many conserved predicted RNA secondary structures and overlapping reading frames (Table 1). (**B**) Schematic representation of IAV mutants with decreased (CpG-low, in blue) and increased (CpG-high, in red) number of CpG motifs. Image was generated with Biorender.

The strategy for generating CpG-high and CpG-low variants of IAV A/WSN/33-wild type (A/WSN/33-WT) consisted of the following steps: (i) The M and NS segments were left unaltered due to the presence of overlapping reading frames and conserved predicted RNA secondary structures throughout the segments (24–26). (ii) Full-length coding sequences of PB2, PB1, PA, and NP from all human IAVs, and HA and NA of H1 and N1 subtypes, isolated between 1900 and 2015, were downloaded from the GISAID EpiFlu database (27) and aligned to A/WSN/33-WT (28, 29). (iii) Genome regions that have been predicted to contain conserved RNA structures and that are involved in packaging or encode proteins in alternative reading frames were excluded from mutagenesis (Table 1 and Fig. 1A). (iv) Only those nucleotide positions that were part of a CpG motif in more than 1% of all viruses but not in A/WSN/33-WT were selected to be modified to increase CpG content. Conversely, CpG motifs naturally absent in more than 1% of all viruses were selected to be modified to decrease CpG content; (v) Only synonymous (silent) mutations were introduced.

**TABLE 1:** Regions in the genome of influenza A virus that were excluded from the CpG mutant design

Segment	Nucleotide position (coding region)	Functions and RNA conserved structure	Reference(s)
PB2	1–132, 2200–2280	Packaging region	(30–32)
	831–960, 1041–1160, 2101–2220	Conserved predicted RNA structure	(26)
PB1	49	Containing start codons for sORF1 and sORF2	(33)
	8–20, 2234–2274	Packaging region	(31, 32)
	51–170, 491–610	Conserved predicted RNA structure	(26, 34)
	95–367	PB1-F2 coding region	(33)
PA	1–66, 2111–2151	Packaging region	(31, 32)
	41–290, 1161–1280, 1611–1860, 1941–2120	Conserved predicted RNA structure	(26)
	453–475	Start site of PA-N155 + 10 nt upstream and downstream	(35)
	534–556	Start site of PA-N182 + 10 nt upstream and downstream	(35)
	568–760	PA-X sequence	(36)
HA	1–45, 1618–1698	Packaging region	(37)
	1–60, 758–808, 961–1080	Conserved predicted RNA structure	(26, 38)
NP	1–120, 1437–1497	Packaging region	(39)
	1–160, 525–555, 441–560, 1031–1250, 1381–1494	Conserved predicted RNA structure	(26, 40)
NA	1–39, 1179–1362	Packaging region	(41)
	531–670	Conserved predicted RNA structure	(26)

Of the 920 potential positions available for silent mutation in the A/WSN/33-WT genome, 258 and 125 positions were modified to create IAV mutants with an increased or decreased number of CpG motifs, resulting in CpG-high with 531 CpG motifs and CpG-low with 148 CpG motifs, respectively (Fig. S1; Fig. 1B and Table 2). This difference of 383 CpG motifs provided a substantial window to assess the impact of altered numbers of CpG motifs in the IAV genome. Despite the extensive mutagenesis, analysis of mononucleotide frequencies revealed comparable proportions of A, U, G, and C in A/WSN/33-WT and the CpG mutant virus segments (Fig. S2). Because synonymous mutations can potentially alter the frequency of other dinucleotides, such as UpA dinucleotides that are also often underrepresented in RNA viruses (42, 43), the relative frequency of UpA dinucleotides was also assessed for the CpG mutants. Despite the substantial differences in the number of CpG motifs, the frequency of UpA dinucleotide motifs remained largely unchanged (Fig. S2).

**TABLE 2:** Composition of CpG motifs per segment in the influenza A virus genome^*a*^

Gene segment	Wild-type virus CpGs (no.)	CpG high virus ΔCpG (no.)	CpG low virus ΔCpG (no.)
PB2	54	+79	−32
PB1	37	+74	−21
PA	43	+30	−13
HA	28	+18	−17
NP	38	+34	−21
NA	32	+23	−21
M & NS^*b*^	41	0	0
Final no. CpGs	273	531	148

^*a*^ ΔCpG, change in the number of CpG motifs.

^*b*^ CpG motifs in the M and NS segments were not modified.

Three independent rescues of (A/WSN/33-WT) and CpG-high and CpG-low mutants were generated, and the presence of the intended mutations was confirmed by next-generation nanopore sequencing for all rescued virus stocks.

### CpG motif content in the IAV genome did not affect virus replication or plaque phenotype

**Fig 2:**
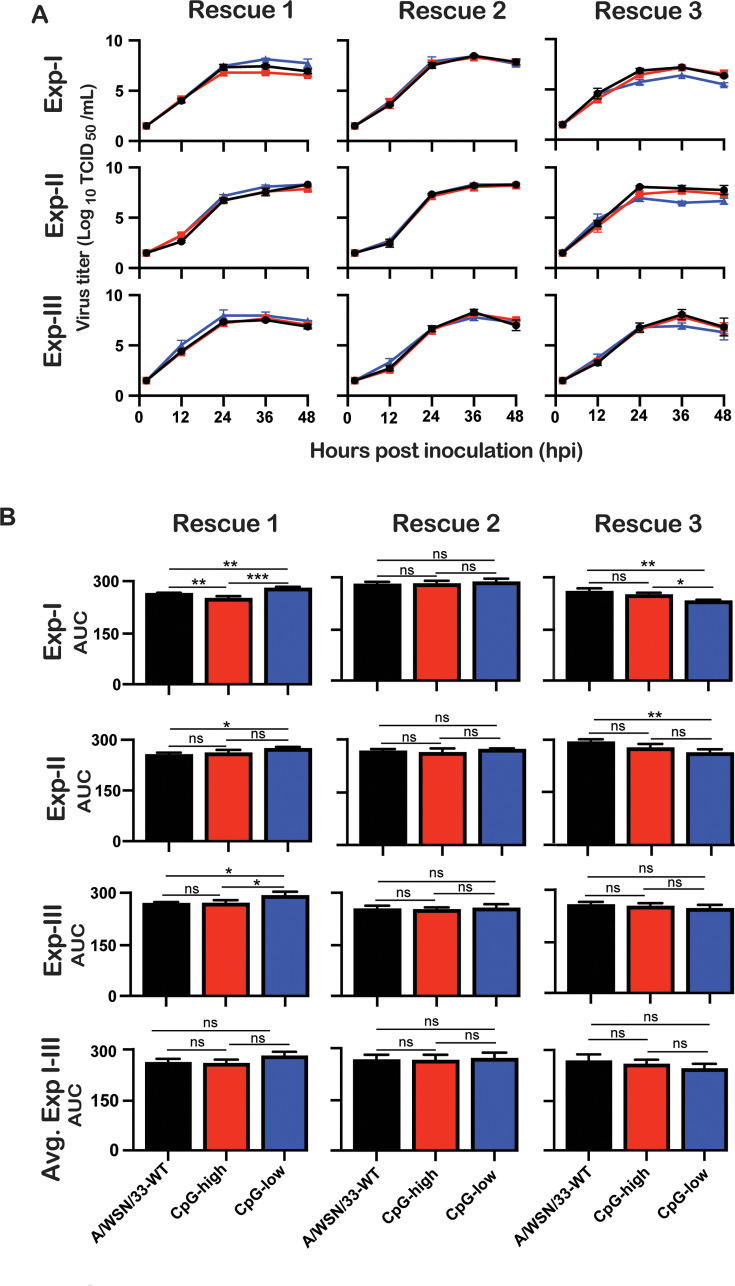
Replication kinetics of CpG-high, CpG-low, and A/WSN/33-WT viruses in MDCK cells. (**A**) MDCK cells were inoculated at an MOI of 0.01, and supernatants were harvested every 12 h for 2 days. Titers (TCID_50_/mL) were determined in MDCK cells. Three independent rescues (1, 2, and 3) were evaluated in three independent experiments (Exp I, II, and III). Red: CpG-high, Blue: CpG-low, and Black: A/WSN/33-WT. (**B**) AUC analysis, calculated for each experiment for all three rescues and the average of AUC of the three experiments for each rescue. Statistical significance was determined by one-way ANOVA with Tukey’s multiple comparison test, where ns, non-significance, and *P  ≤  0.05, **P  ≤  0.01, ***P  ≤  0.001.

To assess whether the number of CpG motifs affected IAV replication, three independent virus rescues of A/WSN/33-WT, CpG-high, and CpG-low were each assessed three times for replication kinetics in MDCK cells. Cells were inoculated at a multiplicity of infection (MOI) of 0.01, and infectious virus titers were determined every 12 h up to 48 hours after inoculation in three independent experiments. Interestingly, despite minor variations, replication kinetics were very similar for A/WSN/33-WT, CpG-high, and CpG-low (Fig. 2). After rescue 1, CpG-high was attenuated slightly compared to CpG-low in one of three replication kinetic experiments but replicated similarly to CpG-low and A/WSN/33-WT in the other two experiments. For rescue 2, there were no differences in replication kinetics between CpG-high, CpG-low, or A/WSN/33-WT in all three replication kinetic experiments. For rescue 3, CpG-high replicated similarly to A/WSN/33-WT and slightly better than or similar to CpG-low in all three replication kinetic experiments (Fig. 2A). Analyses of the area under the curve (AUC) of all three experiments from all three virus rescues confirmed minor variations between the viruses in some experiments but revealed no significant differences between CpG-high, CpG-low, and A/WSN/33-WT for this analysis overall (bottom panel, Fig. 2B).

**Fig 3:**
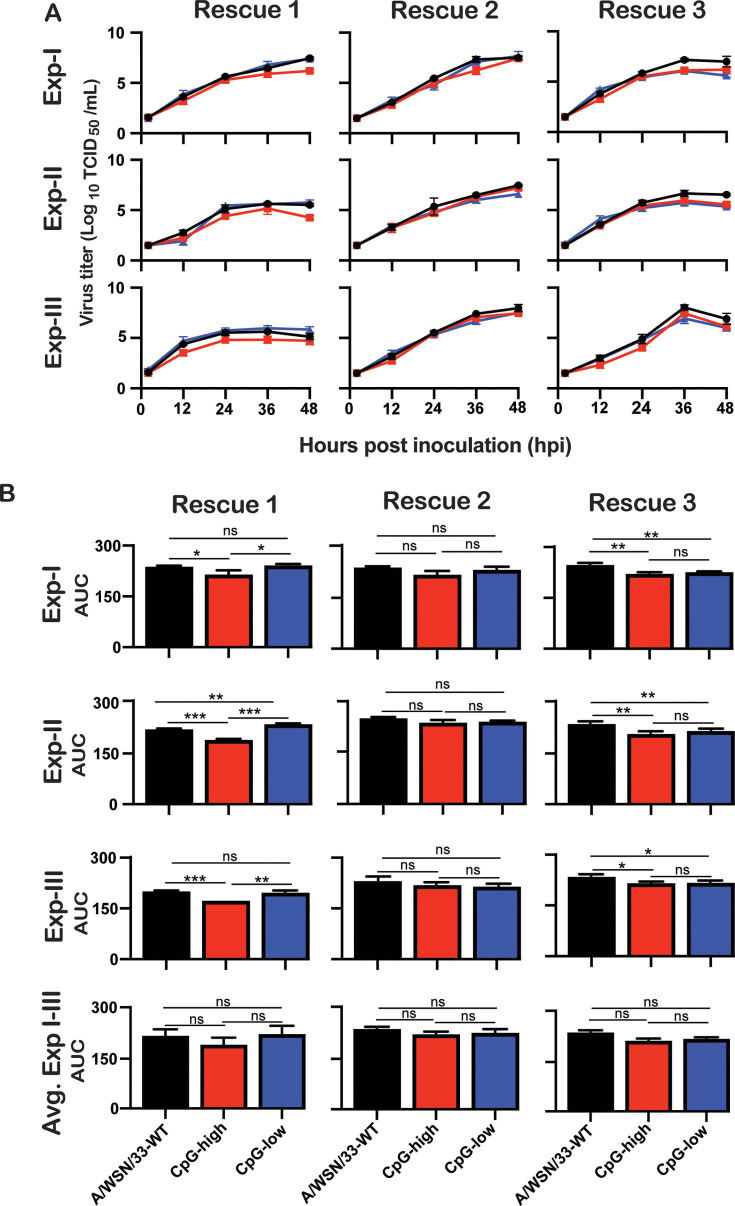
(**A**) Replication kinetics of CpG-high, CpG-low, and A/WSN/33-WT (black) in A549 cells. A549 cells were inoculated at an MOI of 0.01, and supernatants were harvested every 12 h for 2 days. Titers (TCID_50_/mL) were determined in MDCK cells. Three independent rescues (1, 2, and 3) were evaluated in three independent experiments (Exp I, II, and III). Red: CpG-high, Blue: CpG-low, and Black: A/WSN/33-WT. (**B**) AUC analysis, calculated for each experiment for all three rescues and the average of AUC of the three experiments for each rescue. Statistical significance was determined by one-way ANOVA with Tukey’s multiple comparison test, where ns, non-significance, and *P  ≤  0.05, **P ≤ 0.01, ***P ≤ 0.001.

Next, replication kinetics were assessed in immune-competent human respiratory epithelial (A549) cells. Similar to the results obtained in MDCK cells, replication kinetics varied slightly between the different rescues (Fig. 3). For rescue 1, replication of CpG-high was significantly decreased compared to A/WSN/33-WT and CpG-low in all three experiments. No significant differences were observed for virus replication kinetics after rescue 2. The results for rescue 3 were more variable, with similar replication of CpG-low and CpG-high, significantly lower than A/WSN/33-WT in all three experiments. Although small variations were observed for individual viruses from various rescues, analysis of the average AUCs revealed no significant differences between the viruses in the three independent rescues (Fig. 3B).

**Fig 4:**
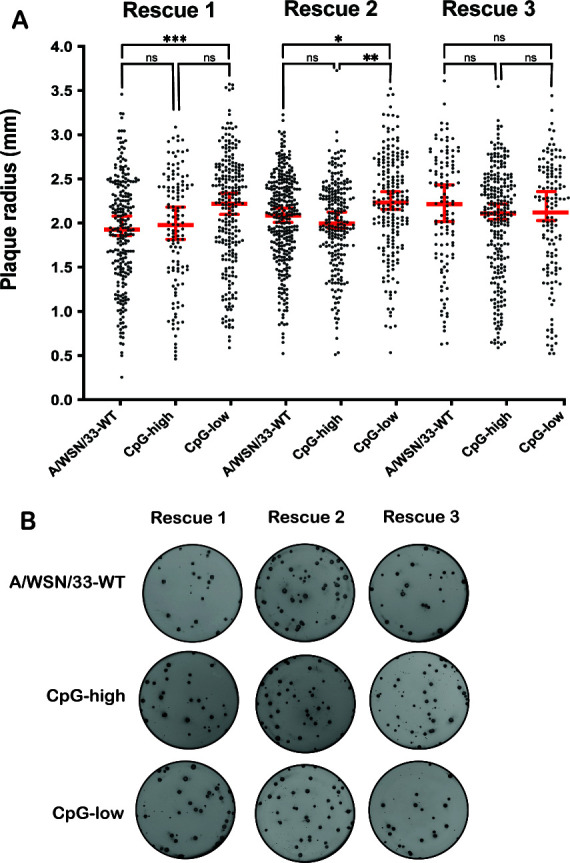
Plaque radius as a measure of virus intrinsic fitness. (**A**) Plaque radii for three independent rescues of A/WSN/33-WT virus and CpG mutants in MDCK cells at 30 hpi. Dots represent individual plaque radius (mm), and the red line represents the median radius (mm) with a 95% confidence interval. Statistical significance was determined using a Kruskal–Wallis test with Dunn’s post hoc correction for multiple comparisons (ns, not significant; *P ≤ 0.05; **P ≤ 0.01; ***P ≤ 0.001). (**B**) Plaque morphology for three independent rescues of wild-type A/WSN/33 and CpG mutants after staining for NP protein at 30 h post-inoculation.

The intrinsic fitness of the CpG mutants was further assessed by plaque assays in MDCK cells using the viruses from the three independent rescues. Consistent with the replication kinetics in MDCK and A549 cells, the average plaque radius varied across rescues (Fig. 4A). Statistically significant differences between CpG-high and CpG-low were observed in rescue 2 but not in rescues 1 and 3. In all three rescues, plaque sizes of the CpG-high and A/WSN/33-WT were comparable. Immunostaining for NP of the plaques further showed that A/WSN/33-WT and the CpG mutants produced plaques with similar morphology (Fig. 4B).

In conclusion, both replication kinetics in MDCK and A549 cells, and plaque assays in MDCK cells demonstrated that a difference of up to 383 CpG motifs between CpG-high, CpG-low, and A/WSN/33-WT had no consistent effect on the virus phenotype, beyond small effects due to experimental variation.

### CpG motif content in the IAV genome did not affect interferon-β induction

**Fig 5:**
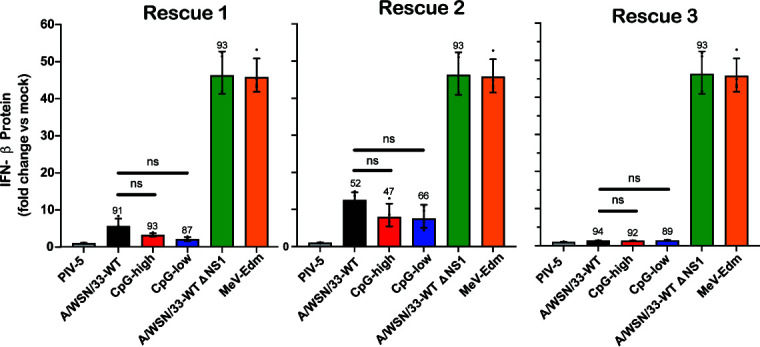
Virus-induced IFN-β production upon inoculation of A549 cells. A549 cells were inoculated at an MOI of 3 with three independent rescues of A/WSN/33-WT (black), CpG-high (red), and CpG-low (blue) virus, and the control viruses, MeV-Edm (yellow), PIV-5 (gray), and A/WSN/33-WT ΔNS1 (green). IFN-β production was measured by luciferase-based bioassay at 24 h post-inoculation. The experiment was performed in triplicate and repeated three times. Numbers above the bars represent the percentage of infected cells measured by FACS. The data shown depict one representative experiment. Statistical significance was determined using a one-way ANOVA with Tukey’s multiple comparison test, where ns means non-significant.

To verify whether the CpG motif underrepresentation in the IAV genome contributes to innate immune evasion, virus-induced activation of the type I Interferon (IFN-β) pathway was assessed. To this end, A549 cells were inoculated at a high MOI of 3 with the three independent rescues of each virus in triplicate. Measles virus strain Edmonston (MeV-Edm) and A/WSN/33-WT-ΔNS1 were included as positive controls, and parainfluenza virus type-5 (PIV-5) as a negative control. Immunostaining for NP confirmed that the percentages of infected cells for the IAV mutants were similar to those observed upon inoculation with A/WSN/33-WT for all three rescues (indicated in Fig. 5 above bars). MeV-Edm and A/WSN/33-WT-ΔNS1 induced levels of IFN-β that were 40-fold higher than those observed upon inoculation with A/WSN/33-WT, whereas CpG-high and CpG-low induced levels, similar to those induced by A/WSN/33-WT, were slightly higher or similar to those for PIV-5. The three viruses of rescue 2 consistently induced higher IFN-β levels than those of the other two rescues, but no significant differences were observed between A/WSN/33-WT and the CpG mutant viruses (Fig. 5). The three rescue 2 viruses consistently induced higher IFN-β levels than those of the other two rescues, but no significant differences were observed between A/WSN/33 and the CpG mutant viruses (Fig. 5).

These findings indicate that a difference of up to 383 CpG motifs between CpG-high and CpG-low and A/WSN/33-WT had no significant effect on virus-induced IFN-β production.

### Introduced CpG motifs exhibit high stability with no evidence for negative selection during serial passaging

To evaluate the stability of the introduced CpG modifications under host cell selection pressure, CpG-high, CpG-low, and A/WSN/33-WT were serially passaged 10 times in A549 and MDCK cells under conditions of limiting dilution. Supernatants from passage 0, 5, and 10 were collected for RNA isolation and full-genome deep sequencing using the Illumina platform, resulting in high coverage across the genome with ~100,000–150,000 reads per virus. Single-nucleotide polymorphism (SNP) analysis with a 10% variant frequency threshold revealed no reversion of the introduced CpG mutations to wild-type sequences. Additionally, no consistent SNP patterns were observed between duplicates of the same virus in either cell line, indicating that the genomes of the CpG mutants remained stable over 10 serial passages (Fig. S3).

**Fig 6:**
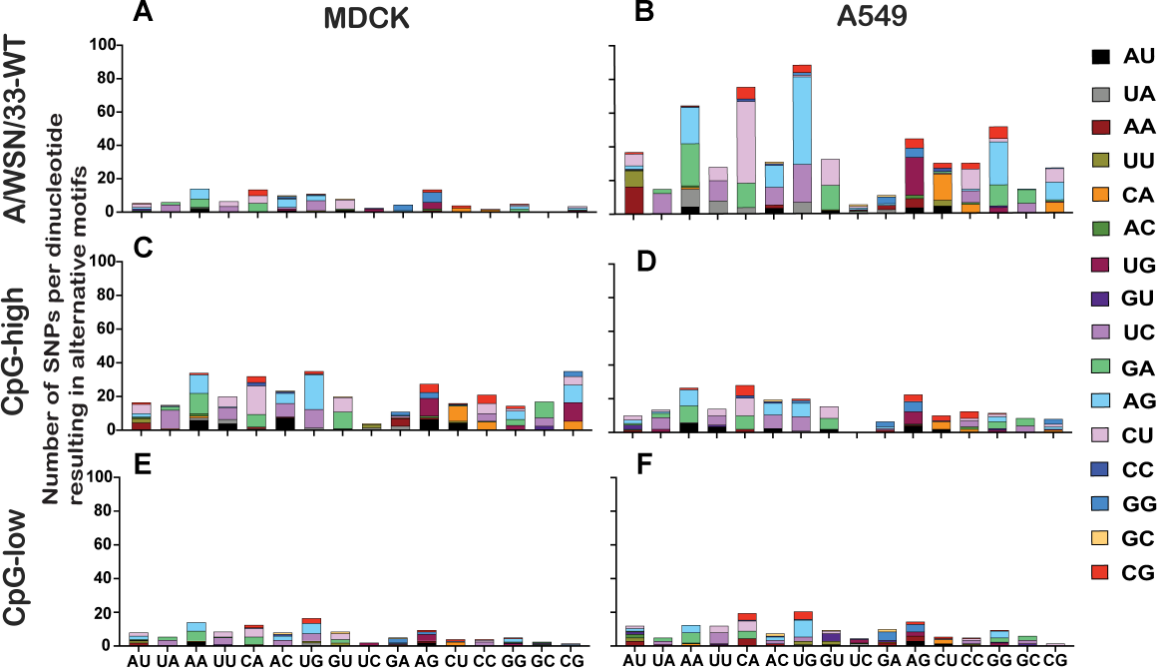
Low-frequency variant-associated changes in dinucleotide motifs during passaging in MDCK (**A, C, and E**) and A549 (**B, D, and F**) cells. Low-frequency variants (>1%) present in passage 10 but absent in passage 0 were analyzed for their impact on dinucleotide motifs across all codon positions; the number of times each motif was converted to an alternative motif is shown. The data represent the average of duplicate experiments. (Fig. S4). The x-axis shows the 16 original dinucleotide contexts, and the y-axis indicates the average number of events that a SNP resulted in a change of that dinucleotide motif to an alternative one. Different colors represent the 16 possible dinucleotide combinations.

To assess whether low-frequency SNPs arising during passaging altered the dinucleotide composition, a more stringent SNP analysis was performed using a 1% variant frequency threshold on viral genome sequences from passage 0 and 10. Positions exceeding this threshold and present exclusively in P10 were used to identify potential changes to alternative dinucleotide motifs across all three codon positions (1-2, 2-3, and 3-1). For each dinucleotide motif, the number of times it was converted into an alternative motif was quantified (Fig. S4 and Fig. 6). Overall, viruses passaged 10 times in A549 cells exhibited slightly higher accumulation of potential changed motifs compared to those passaged 10 times in MDCK cells. A/WSN/33-WT accumulated more SNPs in A549 cells than in MDCK cells, predominantly C-to-U and G-to-A transitions, resulting in the formation of new CpU and ApG dinucleotides (Fig. 6). For CpG-high, the overall mutational pattern was similar in both cell lines. Interestingly, the pressure on CpG dinucleotides to mutate was lower in A549 cells compared to MDCK cells (Fig. 6). Overall, the pressure on CpG dinucleotides to mutate was similar to the pressure on other dinucleotides. For CpG-low, the overall mutational pressure was lower compared to that on A/WSN/33-WT or CpG-high. This pressure was similar between both cell types, with no evidence for preferential restoration of CpG motifs as compared to other dinucleotides (Fig. 6).

Overall, the mutation rate of CpG dinucleotide motif formation or reversion remained below 2% across all viruses in both cell lines and, moreover, the selection pressure on CpG motifs was lower or similar to that on the other 15 dinucleotide motifs in the IAV genome (Fig. 6).

## DISCUSSION

Underrepresentation of CpG motifs in viral genomes is hypothesized to result from host adaptation or evolutionary pressure (8, 44). This has been studied by artificially increasing the number of CpG motifs in specific viral genes, such as those of IAV and echovirus 7 (23, 45). However, most studies either did not compare mutants with high and low CpG content or introduced mutations in only a subset of viral genes. Here, the effect of altered CpG numbers was studied by modifying larger portions of the IAV genome and comparing mutants with either higher or lower numbers of CpG motifs than present in the wild-type virus.

Phenotypic characterization of CpG mutants using three independent rescues and replication kinetics experiments revealed no consistent differences between CpG-high and A/WSN/33-WT, which differ by 258 CpG motifs. More importantly, no consistent differences were observed between CpG-high and CpG-low, which differ by 383 CpG motifs. Variation among the independent rescues could be attributed to differences in the rescue procedure using varying dilutions of 293T transfected supernatants. Rescue 1 was derived from a 1:100 dilution of the 293T supernatant, potentially increasing the chance of accumulation of defective interfering (DI) particles compared to rescues 2 and 3, derived from 1:1,000 and 1:10,000 dilutions, respectively. If DI particles were responsible for the observed variations, increased interferon production or smaller plaque sizes would have been expected for rescue 1 compared to rescues 2 and 3. However, these patterns were not observed, suggesting that the observed variability was not driven by intrinsic properties of the viral stocks.

The results presented here contradict previous reports, showing attenuated replication of IAVs with artificially increased numbers of CpG motifs (22, 23, 42). These differences are likely due to variation in the strategies used to generate CpG mutants. For instance, Guant et al. introduced mutations restricted to specific gene segments, such as the NP gene segment of IAV (23), whereas in our study, silent mutations were introduced across six of the eight IAV genes. By altering CpG numbers in multiple segments, gene-specific biases were avoided while increasing the number of CpG motifs substantially. Other studies achieved extremely high numbers of CpG motifs by adding CpG motifs at all possible positions in a given gene segment while including compensatory mutations to maintain the mononucleotide frequency. In these designs, conserved RNA regions (such as important RNA structures) were not always considered, and crucial elements important for virus replication could have been changed.

In our study, the CpG mutants were generated by introducing mutations at positions that are part of CpG motifs in more than 1% of circulating IAV strains but are absent in A/WSN/33-WT. By selecting naturally occurring positions, mutations were restricted to sites tolerated by the viruses without loss of fitness. In contrast, other studies did not restrict CpG introduction to naturally occurring positions and may therefore have included sites rarely modified in nature, possibly due to functional constraints. Although no single virus carries all CpG-high mutations simultaneously, our constructs combined these tolerated positions, creating the maximum difference in CpG motifs between CpG-high and CpG-low. Given these maximal differences, the absence of attenuation does not seem to result from the selection of naturally occurring sites. Consequently, the observed attenuation in other studies may reflect the design strategy rather than the direct effect of increased CpG numbers. In support of this, a recent study by Sharp et al. reported that introducing two compensatory mutations in the NP segment of IAV, aimed at maintaining mononucleotide frequency, attenuated virus replication compared to wild-type virus (21).

Attenuation resulting from codon pair deoptimization is often misattributed to an increased number of CpG motifs, as CpG motifs located at codon pair boundaries (NNC-p-GNN) are among the most underrepresented in vertebrate genomes (46). Although a few studies have aimed to distinguish the effect of codon pair deoptimization from those of increased CpG numbers, the results have often been contradictory (47–50). Groenke et al. carefully designed two IAV NA mutants, one with an increased CpG number and another with suboptimal codon pair deoptimization. They reported that increasing CpG numbers did not attenuate the virus, whereas suboptimal codon pair deoptimization did (46). Our findings on the absence of phenotypic changes of CpG-high and CpG-low are consistent with these observations. Of note, our study did not specifically control for the potential confounding effects of codon pair deoptimization versus changes in CpG motif frequency. While we cannot exclude the possibility that UpA content contributed to phenotypes observed in other studies, including Gaunt et al., who reported that viruses with elevated UpA were attenuated, in our study, UpA frequencies were unaltered. These observations support the conclusion that CpG enrichment alone does not necessarily alter viral phenotype.

It has been shown that the ZAP specifically binds to CpG motifs in the HIV-1 genome (18). The role of ZAP has also been studied for the recognition of CpG motifs in the genomes of echovirus 7, Zika virus, human cytomegalovirus, and IAV (17, 42, 51–53). In most studies, the attenuation observed for CpG-enriched viruses was reversed in ZAP knockout cells (18, 42, 51, 53, 54). However, an opposite observation was reported for HIV-1 CpG high, where no role for ZAP was detected(20). We confirmed ZAP expression in our parental A549 cells, and assessment of replication kinetics in ZAP knock-out (KO) A549 cells revealed no significant phenotypic differences between the WT and CpG-high viruses, either in the presence or absence of ZAP (Fig. S5). These findings are consistent with Sharp et al. (21), who reported that their CpG-high IAV was not attenuated by ZAP. Surprisingly, the CpG-low virus exhibited a modest attenuation in ZAP-KO cells relative to wild-type cells. This observation is not consistent with the hypothesis that reduced CpG content decreases ZAP-mediated restriction and would therefore enhance replication in the absence of ZAP. While increased CpG content has been associated with reduced viral replication through ZAP-mediated restriction, a decrease in CpG frequency would not be expected to impair replication under this model. The attenuation of the CpG-low virus in ZAP-KO cells is therefore not readily explained by a CpG-dependent mechanism.

Luo et al. (55) reported that ZAP recognition depends not only on CpG frequency but also on RNA structure, as CpG motifs must be accessible in single-stranded regions for efficient binding, suggesting that the location and spacing of CpG motifs in our mutants may represent an additional explanation for the absence of ZAP-mediated attenuation.

Goncalves et al. reported that spacing CpG nucleotides 14 nucleotides apart in an AU-rich context may constitute a ZAP-response element (ZRE) in viral RNA, increasing ZAP sensitivity (56). In our study, the average distance between CpG motifs in CpG-high was ~15 nucleotides, yet no significant attenuation in replication kinetics was observed. It therefore remains unclear whether a threshold level, specific clustering, or structural context of CpGs is required for ZAP recognition. RNA secondary structures in viruses not only help host cells differentiate between self and non-self (57, 58), but also play an important role in IAV replication and packaging (59–62). Disrupting these structures can be detrimental to virus replication (24, 25, 40, 60, 61), which may explain why some studies observed attenuation of CpG-high viruses. For example, Sharp et al. designed CpG-high mutants by densely adding CpG motifs throughout the PB2 segment of influenza virus A/Puerto Rico/8/1934 without accounting for all secondary structures. Notably, CpG motifs were added in the region spanning nucleotides 831–960 and 1041–1160 of the PB2 segment that were excluded in our study due to their predicted secondary structures (26). Moreover, disruption of RNA secondary structures could increase the sensitivity of viral RNA to antiviral proteins. In support of this, Chiu et al. reported that ZAP mainly targets the CpG-rich dumbbell structure in the 3'-untranslated region (3′-UTR) of Japanese encephalitis virus (JEV) and suggested that small differences in potential ZAP-binding sites and viral RNA structures can affect ZAP sensitivity (63). These findings highlight the critical role of RNA secondary structures in shaping selection pressure on synonymous substitutions.

It is important to note that the design strategy of the CpG mutants was based on IAV RNA structures, wherein the structural constraints imposed by their functions were the most evident, having been known already about ten years ago. More recently, information on genome-wide IAV RNA secondary structures of several strains, including A/WSN/33-WT, has been elucidated by modern RNA structure probing methods (60, 61, 64). However, many elements of these genomic structures are not conserved between different strains and are likely to have no or redundant functions.

If the CpG mutants were designed today, the number and placement of CpG motifs might differ slightly, potentially reducing the number of CpG motifs in CpG-high and CpG-low. However, it seems unlikely that altering a few additional CpG motifs would lead to more significant effects than those observed in our study, which compared viruses differing by 379 CpG motifs and yielded no substantial phenotypic differences.

Consistent with previous studies, no significant differences were observed in virus-induced IFN production between CpG-high and CpG-low or A/WSN/33-WT across three independent rescues (21, 22). This suggests that changing the number of CpG motifs either does not affect activation of the IFN-β pathway or that the IFN antagonist NS1 (65) is so effective in suppressing this response that any minor differences caused by the altered number of CpG motifs are undetectable. Notably, Sharp et al. did not observe significant differences in IFN induction when using IAV CpG mutants harboring functional mutations in the NS1 gene (22). These findings support that CpG motifs do not play a role in activating the IFN pathway even in the absence of NS1.

Our data from serial passaging and subsequent genome sequencing revealed that the introduced CpG motifs remained largely stable even after ten passages in two different cell lines. Moreover, 1% SNP variant analysis indicated that the selection pressure on CpG motifs was lower or comparable to that on other dinucleotide motifs. These data suggest that CpG motifs are not subject to strong host selection pressure *in vitro*, consistent with previous reports (22).

Although this study was conducted with the lab-adapted A/WSN/33 virus, the introduced CpG motifs are also present in naturally circulating human viruses across all subtypes. Therefore, it is likely that similar results would be observed with other IAV strains.

Many studies have explored codon usage bias, codon-pair bias, and dinucleotide bias, as well as relationships between them (13, 48, 66–68). However, the context of mononucleotide bias in the viral genome is often overlooked, even though the unusual nucleotide composition of RNA viruses was recognized a long time ago (9, 69). van Hemert et al. analyzed a broad range of RNA virus genomes and highlighted the significance of nucleotide composition in the context of RNA structure, concluding that mononucleotide bias is the major driving force behind virus-specific codon usage. Moreover, analysis of single-stranded RNA genomes has revealed a general trend of A-rich and C-poor sequences in predicted RNA structures across the majority of RNA viruses (70). It is possible that the CpG underrepresentation is an artifact of the mono-nucleotide bias, resulting from natural selection to maintain RNA structural integrity. Further controlled studies are needed to better understand the complex interplay between mononucleotide bias, dinucleotide bias, and RNA secondary structures.

Recently, CpG enrichment has gained attention as a strategy for designing live-attenuated vaccines (22, 71). By carefully designing and comparing IAV mutants with increased or decreased numbers of CpG motifs, using three biological replicates with three experimental repeats, no significant phenotypic effects were observed. Since the underlying reason for CpG underrepresentation in RNA viral genomes remains unclear, strategies for designing live-attenuated vaccines based on CpG enrichment should, at a minimum, consider RNA secondary structures and conserved regions.

## MATERIALS AND METHODS

### Mammalian cell culture

Madin-Darby canine kidney (MDCK) cells were cultured in Eagle’s minimal essential medium (EMEM, Capricorn Scientific) with 1.5 mg/mL of sodium bicarbonate, 20 mM HEPES, and 1% Nonessential amino acid solution (NEAA, Lonza). Human respiratory epithelial (A549) cells were cultured in Ham’s F12 medium (Capricorn Scientific). Human embryonic kidney (HEK-293T) cells were cultured in Dulbecco’s modified Eagle medium (DMEM) with high glucose (4.5 g/L) and supplemented with 2 mM L-Glutamine (Capricorn Scientific), 1 mM sodium pyruvate (Gibco), 0.5 mg/mL geneticin (Invitrogen), and 1% Nonessential amino acids (NEAA, Lonza). All cell culture media were supplemented with 10% fetal bovine serum (FBS, Sigma-Aldrich), and a 100 U/mL penicillin–streptomycin–glutamine mixture (Capricorn) and cultured at 37°C with 5% CO_2_. All cell lines used in this study tested negative for the presence of mycoplasma and were maintained at low passage numbers.

### Virus rescue

IAV A/Wilson-Smith/1933 H1N1 (A/WSN/33-WT) was used to generate mutants with increased or decreased numbers of CpG motifs (72). CpG motifs were introduced at the level of the cDNA rescue plasmids. During virus rescue, these plasmids generate viral RNA, meaning that the introduced mutations are incorporated into the genomic vRNA and consequently also present in the derived cRNA and mRNA. Genes containing the designed mutations in PB2, PB1, PA, HA, NP, and NA were synthesized at Integrated DNA Technologies and ProteoGenix. The synthetic fragments were subsequently PCR amplified and cloned into the A/WSN/33 bidirectional pHW2000 vector, a gift from Dr. Webster (St. Jude Children’s Research Hospital, Memphis, TN, USA). The mutants were rescued as described previously (73). In brief, HEK-293T cells were transfected with six segments, PB2, PB1, PA, HA, NP, and NA, of A/WSN/33 or CpG-mutants together with the two unmodified M and NS segments. After 72 h, varying dilutions of 293T supernatants (1:100, 1:1,000, or 1:10,000) were used to inoculate MDCK cells in EMEM medium, in the presence of 1.6 µg/mL TPCK trypsin (Merck) to produce three different viral rescues (rescues 1, 2, and 3, respectively). At 36 h after inoculation, upon reaching 70% cytopathic effect, viruses were harvested. A/WSN/33-WT-ΔNS1 was generated by deleting the NS1 ORF and rescuing in MDCK-NS1 cells (a kind gift from Adolfo Gracia-Sastre). Measles virus strain Edmonston (MeV-Edm) and PIV-5 strain W3 were generated as described previously (74–76). Viral titrations were performed as previously described (77), and the infectious titer (TCID_50_/mL) was calculated using the Spearman-Karber method (78).

### Nanopore sequencing of viral genomes

To confirm that the viral genomes contained the intended mutations, viral RNA was isolated from virus stocks using the High Pure RNA Isolation Kit (Roche) according to the manufacturer’s instructions. Viral RNA was then used to amplify all 8 segments of the IAV genome using Uni-12 and Uni-13 primers and Superscript III one-step RT-PCR system with Platinum Taq High Fidelity DNA Polymerase (Invitrogen) (79). Cycling conditions were 55°C for 2 min, 42°C for 60 min, 94°C for 2 min, followed by 5 cycles of (94°C for 30 s; 44°C for 30 s; 68°C for 3.5 min) and 35 cycles of (94°C for 30 s, 57°C for 30 s; 68°C for 3.5 min) and finally 68°C for 10 min. Amplified viral cDNA was purified using Agencount AMPure XP beads (Beckman Coulter). Libraries were generated using the Oxford Nanopore ligation sequencing kit (SQK-LSK109, Oxford Nanopore) and sequenced in an R9.4 flow cell (Oxford Nanopore). The sequencing reads were demultiplexed using the Porechop Script and analyzed using CLC Genomics Workbench (Qiagen, version 20) (80).

### Viral replication kinetics

For replication kinetics, monolayers of 2 × 10^6^ A549 or MDCK cells were seeded in six-well plates 24 h before inoculation. The cells were inoculated in triplicate at an MOI of 0.01 for 2 h at 37°C. Thereafter, cells were washed three times with phosphate-buffered saline (PBS) and replenished with the MDCK infection media consisting of complete EMEM media without FCS and with 1.6 µg/mL TPCK-trypsin for MDCK cells, or a combination of 0.1% FBS and 0.6 µg/mL TPCK-trypsin in Ham’s F12 medium for A549 cells, as these cells are more sensitive to trypsin. Supernatants were collected every 12 h and stored at −80°C until titration.

### Plaque assays

For plaque assays, 2 × 10^6^ MDCK cells were seeded in six-well plates to obtain a 90% confluent monolayer the next day. Virus stocks were 10-fold serially diluted until a concentration that produced approximately 15 plaques per well. Cells were inoculated with the optimal virus dilution in 1 mL of infection media and incubated for 1 h at 37°C. After incubation, the cells were washed with PBS, and 4 mL of an overlay containing a 1:1 mixture of 2× EMEM (Capricorn Scientific) with all the components of MDCK infection media and Avicel (IMCD) was added to the cells. Plates were incubated for 30 h at 37°C and 5% CO2. After 30 h, the plates were washed with PBS twice and fixed with 80% acetone in PBS at −20°C for a minimum of 3 h. Virus infection was detected by immunostaining with an IAV NP monoclonal antibody (1:1,000, IgG2a, clone Hb65, ATCC) and goat-anti-mouse Ig FITC antibody (1:100; BD Biosciences). The stained plaques were scanned using a Typhoon scanner (GE Healthcare), and the plaque radius was measured using Image QuantTL software.

### Serial passaging

Viruses were serially diluted in 10-fold steps until dilution 10^−10^, after which A549 and MDCK cells were inoculated in a 12-well plate in duplicate. After 2 days, HA assays were performed to detect the virus in the culture supernatants. The highest dilution with a positive HA titer (≥4) was used as inoculum for the next passage, for a total of 10 passages. Finally, the viruses in the supernatant from passages 0, 1, 5, and 10 were subjected to next-generation Illumina sequencing.

### IFN-β bioassay and FACS analysis

To detect IFN-β production, 2 × 10^5^ A549 cells in 24-well plates were inoculated at an MOI of 3. At 24 h post-inoculation, the supernatants were collected for ISRE-firefly luciferase reporter assays (81), and cells were harvested for FACS to determine the infection percentage. For FACS, cells were fixed and permeabilized with BD Cytofix/Cytoperm (BD Bioscience), stained with NP antibody (1:100; IgG2a, clone Hb65, ATCC) in BD Perm/Wash buffer, followed by staining with Alexa Fluor 488 conjugated goat anti-mouse IgG2α antibody (1:100; Thermo-Fisher). The percentage of NP-positive cells was determined using the FlowJo v10.7.2 software (BD Biosciences).

### llumina sequencing

Viral RNA was extracted using the High Pure RNA Isolation Kit (Roche), and all eight gene influenza segments were amplified using Uni-12′ and Uni-13′ specific primers as described in nanopore sequencing. Amplified viral cDNA was purified using Agencourt AMPure XP beads and subjected to an enzymatic fragmentation step for 10 min to produce DNA fragments of 300–400 bp. Sequencing libraries were generated using the KAPA Library Quantification Kit (Roche) and the KAPA Unique Dual-Indexed Adapter Kit (Roche), according to the manufacturer’s protocol (Roche, SeqCap EZ HyperCap Workflow, version 2.2). Libraries were pooled in equimolar ratios and sequenced to generate paired-end 300 bp reads using a 600-cycle Miseq V3 reagent kit (Illumina, MISEQ 2023 062). After sequencing, demultiplexed fastq reads were mapped to references and consensuses, and a minimum coverage of 100 reads per nucleotide was extracted. Variant analysis was performed using the Basic-Variant detection program in CLC Genomics Workbench (Qiagen, version 20) (82).

### Statistical analysis

Statistical analyses were performed using GraphPad Prism 10.4.0 (GraphPad Software Inc., version 10.6.1). For virus replication kinetics data, the AUC was calculated for each experiment, and the average of all three independent experiments from each rescue. Statistical significance was determined using a one-way ANOVA with Tukey’s multiple comparison test or Kruskal–Wallis test with Dunn’s post hoc correction for multiple comparisons.

## Data Availability

The original data were uploaded into the European Nucleotide Archive with the project accession code PRJEB112293 and secondary accession code ERP192871, with run file accession codes ERR17157572–ERR17157584 for CpG-high virus, ERR17157585–ERR17157597 for CpG-low virus, and ERR17157598–ERR17157610 for WT virus.

## SUPPLEMENTAL MATERIAL

Fig. S1 jvi.00047-26-s0001.docx Nucleotide sequences of WT and mutant viruses.

Fig. S2 jvi.00047-26-s0002.docx Mono-and di-nucleotide frequencies of WT and mutant viruses.

Fig. S3 jvi.00047-26-s0003.xlsx Table of SNPs with >1% mutation frequency.

Fig. S4 jvi.00047-26-s0004.xlsx Change of dinucleotide motifs upon passaging.

Fig. S5 jvi.00047-26-s0005.tif Role of ZAP in recognition of IAV CpG mutants.

Supplemental legends jvi.00047-26-s0006.docx Descriptive legends for supplemental material.

ASM does not own the copyrights to Supplemental Material that may be linked to, or accessed through, an article. The authors have granted ASM a non-exclusive, world-wide license to publish the Supplemental Material files. Please contact the corresponding author directly for reuse.
