# Supplementary material for: Increased or decreased numbers of CpG dinucleotide motifs in the genome of influenza A virus do not affect in vitro virus phenotype
Source: J Virol. 2026 Jun 22;100(7):e00047-26. doi: 10.1128/jvi.00047-26 (PMC13386996; doi:10.1128/jvi.00047-26)
Supplement: Fig. S1 — Nucleotide sequences of WT and mutant viruses. [file jvi.00047-26-s0001.docx]

**Fig S1: Nucleotide sequence of A) A/WSN/33-WT, B) CpG-High and C) CpG-low,**

**sequences given per segment**

**A) A/WSN/33-WT**

>WSN_WT_PB2

ATGGAAAGAATAAAAGAACTAAGGAATCTAATGTCGCAGTCTCGCACTCGCGAGATACTCACAAAAACCACCGTGGACCATATGGCCATAATCAAGAAGTACACATCAGGAAGACAGGAGAAGAACCCAGCACTTAGGATGAAATGGATGATGGCAATGAAATATCCAATTACAGCAGACAAGAGGATAACGGAAATGATTCCTGAGAGAAATGAGCAGGGACAAACTTTATGGAGTAAAATGAATGACGCCGGATCAGACCGAGTGATGGTATCACCTCTGGCTGTGACATGGTGGAATAGGAATGGACCAGTGACAAGTACAGTTCATTATCCAAAAATCTACAAAACTTATTTTGAAAAAGTCGAAAGGTTAAAACATGGAACCTTTGGCCCTGTCCATTTTAGAAACCAAGTCAAAATACGTCGAAGAGTTGACATAAATCCTGGTCATGCAGATCTCAGTGCCAAAGAGGCACAGGATGTAATCATGGAAGTTGTTTTCCCTAACGAAGTGGGAGCCAGGATACTAACATCGGAATCGCAACTAACGACAACCAAAGAGAAGAAAGAAGAACTCCAGGGTTGCAAAATTTCTCCTCTGATGGTGGCATACATGTTGGAGAGAGAACTGGTCCGCAAAACGAGATTCCTCCCAGTGGCTGGTGGAACAAGCAGTGTGTACATTGAAGTGTTGCATTTGACCCAAGGAACATGCTGGGAACAGATGTACACTCCAGGAGGGGAGGCGAGGAATGATGATGTTGATCAAAGCTTAATTATTGCTGCTAGAAACATAGTAAGAAGAGCCACAGTATCAGCAGATCCACTAGCATCTTTATTGGAGATGTGCCACAGCACGCAGATTGGTGGAATAAGGATGGTAAACATCCTTAGGCAGAACCCAACAGAAGAGCAAGCCGTGGATATTTGCAAGGCTGCAATGGGACTGAGAATTAGCTCATCCTTCAGTTTTGGTGGATTCACATTTAAGAGAACAAGCGGATCATCAGTCAAGAGAGAGGAAGAGGTGCTTACGGGCAATCTTCAGACATTGAAGATAAGAGTGCATGAGGGATATGAAGAGTTCACAATGGTTGGGAGAAGAGCAACAGCTATACTCAGAAAAGCAACCAGGAGATTGATTCAGCTGATAGTGAGTGGGAGAGACGAACAGTCGATTGCCGAAGCAATAATTGTGGCCATGGTATTTTCACAAGAGGATTGTATGATAAAAGCAGTTAGAGGTGACCTGAATTTCGTCAATAGGGCGAATCAGCGATTGAATCCCATGCACCAACTTTTGAGACATTTTCAGAAGGATGCAAAGGTGCTCTTTCAAAATTGGGGAATTGAATCCATCGACAATGTGATGGGAATGATCGGGATATTGCCCGACATGACTCCAAGCACCGAGATGTCAATGAGAGGAGTGAGAATCAGCAAAATGGGGGTAGATGAGTATTCCAGCGCGGAGAAGATAGTGGTGAGCATTGACCGTTTTTTGAGAGTTAGGGACCAACGTGGGAATGTACTACTGTCTCCCGAGGAGATCAGTGAAACACAGGGAACAGAGAAACTGACAATAACTTACTCATCGTCAATGATGTGGGAGATTAATGGTCCTGAATCAGTGTTGGTCAATACCTATCAGTGGATCATCAGAAACTGGGAAACTGTTAAAATTCAGTGGTCCCAGAATCCTACAATGCTGTACAATAAAATGGAATTTGAGCCATTTCAGTCTTTAGTTCCAAAGGCCGTTAGAGGCCAATACAGTGGGTTTGTGAGAACTCTGTTCCAACAAATGAGGGATGTGCTTGGGACATTTGATACCGCTCAGATAATAAAACTTCTTCCCTTCGCAGCCGCTCCACCAAAGCAAAGTAGAACGCAGTTCTCCTCATTGACTATAAATGTGAGGGGATCAGGAATGAGAATACTTGTAAGGGGCAATTCTCCAGTATTCAACTACAACAAGACCACTAAAAGACTCACAGTTCTCGGAAAGGATGCTGGCCCTTTAACTGAAGACCCAGATGAAGGCACAGCTGGAGTTGAGTCCGCAGTTCTGAGAGGATTCCTCATTCTGGGCAAAGAAGACAGGAGATATGGACCAGCATTAAGCATAAATGAACTGAGCAACCTTGCGAAAGGAGAGAAGGCTAATGTGCTAATTGGGCAAGGAGACGTGGTGTTGGTAATGAAACGGAAACGGAACTCTAGCATACTTACTGACAGCCAGACAGCGACCAAAAGAATTCGGATGGCCATCAATTAGTGTCGAATAGTTTAAAAACGACCTTGTTTCTACT

>WSN_WT_PB1

ATGGATGTCAATCCGACTTTACTTTTCTTAAAAGTGCCAGCACAAAATGCTATAAGCACAACTTTCCCTTATACTGGAGACCCTCCTTACAGCCATGGGACAGGAACAGGATACACCATGGATACTGTCAACAGGACACATCAGTACTCAGAAAGGGGAAGATGGACAACAAACACCGAAACTGGAGCACCGCAACTCAACCCGATTGATGGGCCACTGCCAGAAGACAATGAACCAAGTGGTTATGCCCAAACAGATTGTGTATTGGAAGCAATGGCCTTCCTTGAGGAATCCCATCCTGGTATCTTTGAGACCTCGTGTCTTGAAACGATGGAGGTTGTTCAGCAAACACGAGTGGACAAGCTGACACAAGGCCGACAGACCTATGACTGGACTCTAAATAGGAACCAGCCTGCTGCAACAGCATTGGCCAACACAATAGAAGTGTTCAGATCAAATGGCCTCACGGCCAATGAATCTGGAAGGCTCATAGACTTCCTTAAGGATGTAATGGAGTCAATGAACAAAGAAGAAATGGAGATCACAACTCATTTTCAGAGAAAGAGACGAGTGAGAGACAATATGACTAAGAAAATGGTGACACAGAGAACAATAGGTAAAAGGAAGCAGAGATTGAACAAAAGGAGTTATCTAATTAGGGCATTAACCCTGAACACAATGACCAAAGATGCTGAGAGAGGGAAGCTAAAACGGAGAGCAATTGCAACCCCAGGGATGCAAATAAGGGGGTTTGTATACTTTGTTGAGACACTAGCAAGGAGTATATGTGAGAAACTTGAACAATCAGGATTGCCAGTTGGAGGCAATGAGAAGAAAGCAAAGTTGGCAAATGTTGTAAGGAAGATGATGACCAATTCTCAGGACACTGAAATTTCTTTCACCATCACTGGAGATAACACCAAATGGAACGAAAATCAGAACCCTCGGATGTTTTTGGCCATGATCACATATATAACCAGAAATCAGCCCGAATGGTTCAGAAATGTTCTAAGTATTGCTCCAATAATGTTCTCAAACAAAATGGCGAGACTGGGAAAGGGGTACATGTTTGAGAGCAAGAGTATGAAAATTAGAACTCAAATACCTGCAGAAATGCTAGCAAGCATCGATTTGAAATACTTCAATGATTCAACTAGAAAGAAGATTGAAAAAATCCGGCCGCTCTTAATAGATGGGACTGCATCATTGAGCCCTGGAATGATGATGGGCATGTTCAATATGTTAAGTACTGTATTAGGCGTCTCCATCCTGAATCTTGGACAAAAGAGACACACCAAGACTACTTACTGGTGGGATGGTCTTCAATCTTCTGATGATTTTGCTCTGATTGTGAATGCACCCAATCATGAAGGGATTCAAGCCGGAGTCAACAGGTTTTATCGAACCTGTAAGCTACTTGGAATTAATATGAGCAAGAAAAAGTCTTACATAAACAGAACAGGTACATTTGAATTCACAAGTTTTTTCTATCGTTATGGGTTTGTTGCCAATTTCAGCATGGAGCTTCCCAGCTTTGGGGTGTCTGGGATCAACGAGTCTGCGGACATGAGTATTGGAGTTACTGTCATCAAAAACAATATGATAAACAATGATCTTGGTCCAGCAACCGCTCAAATGGCCCTTCAGCTGTTCATCAAAGATTACAGGTACACGTACCGGTGCCATAGAGGTGACACACAAATACAAACCCGAAGATCATTTGAAATAAAGAAACTGTGGGAGCAAACCCATTCCAAAGCTGGACTGCTGGTCTCCGACGGAGGCCCAAATTTATACAACATTAGAAATCTCCACATTCCTGAAGTCTGCTTGAAATGGGAATTAATGGATGAGGATTACCAGGGGCGTTTATGCAACCCACTGAACCCATTTGTCAACCATAAAGACATTGAATCAGTGAACAATGCAGTGATAATGCCAGCACATGGTCCAGCCAAAAACATGGAGTATGATGCTGTTGCAACAACACACTCCTGGATCCCCAAAAGAAATCGATCCATCTTGAATACAAGCCAAAGAGGAATACTTGAAGATGAACAAATGTACCAAAAGTGCTGCAACTTATTTGAAAAATTCTTCCCCAGCAGTTCATACAGAAGACCAGTCGGGATATCCAGTATGGTGGAGGCTATGGTTTCCAGAGCCCGAATTGATGCACGAATTGATTTCGAATCTGGAAGGATAAAGAAAGAGGAGTTCACTGAGATCATGAAGATCTGTTCCACCATTGAAGAGCTCAGACGGCAAAAATAGTGAATTTAGCTTGTCCTTCATGAAAAAATGCCTTGTTTCTACT

>WSN_WT_PA

ATGGAAGATTTTGTGCGACAATGCTTCAATCCGATGATTGTCGAGCTTGCGGAAAAGGCAATGAAAGAGTATGGAGAGGACCTGAAAATCGAAACAAACAAATTTGCAGCAATATGCACTCACTTGGAAGTGTGCTTCATGTATTCAGATTTTCACTTCATCGATGAGCAAGGCGAGTCAATAGTCGTAGAACTTGGCGATCCAAATGCACTTTTGAAGCACAGATTTGAAATAATCGAGGGAAGAGATCGCACAATAGCCTGGACAGTAATAAACAGTATTTGCAACACTACAGGGGCTGAGAAACCAAAGTTTCTACCAGATTTGTATGATTACAAGAAGAATAGATTCATCGAAATTGGAGTAACAAGGAGAGAAGTTCACATATACTATCTGGAAAAGGCCAATAAAATTAAATCTGAGAAGACACACATCCACATTTTCTCATTCACTGGGGAGGAAATGGCCACAAAGGCCGACTACACTCTCGATGAAGAAAGCAGGGCTAGGATCAAAACCAGGCTATTCACCATAAGACAAGAAATGGCTAGCAGAGGCCTCTGGGATTCCTTTCGTCAGTCCGAGAGAGGCGAAGAGACAATTGAAGAAAGATTTGAAATCACAGGAACAATGCGCAAGCTTGCCGACCAAAGTCTCCCGCCAAACTTCTCCAGCCTTGAAAAATTTAGAGCCTATGTGGATGGATTCGAACCGAACGGCTACATTGAGGGCAAGCTTTCTCAAATGTCCAAAGAAGTAAATGCTAGAATTGAACCTTTTTTGAAATCAACACCACGACCACTTAGACTTCCGGATGGGCCTCCCTGTTCTCAGCGGTCCAAATTCCTGCTGATGGATGCCTTAAAATTAAGCATTGAGGACCCAAGTCATGAGGGAGAGGGGATACCGCTATATGATGCAATCAAATGCATGAGAACATTCTTTGGATGGAAGGAACCCAATGTTGTTAAACCACACGAAAAGGGAATAAATCCAAATTATCTTCTGTCATGGAAGCAAGTACTGGCAGAACTGCAGGACATTGAGAATGAGGAGAAAATTCCAAGGACTAAAAATATGAAGAAAACGAGTCAGTTAAAGTGGGCACTTGGTGAGAACATGGCACCAGAAAAGGTAGACTTTGACGATTGTAAAGATGTAGGCGATTTGAAGCAATATGATAGTGATGAACCAGAATTGAGGTCGCTTGCAAGTTGGATTCAGAATGAGTTCAACAAGGCATGTGAACTGACCGATTCAAGCTGGATAGAGCTCGATGAGATTGGAGAAGATGCGGCTCCAATTGAACACATTGCAAGCATGAGAAGGAATTATTTCACAGCAGAGGTGTCTCATTGCAGAGCCACAGAATACATAATGAAGGGGGTGTACATCAATACTGCCTTGCTTAATGCATCCTGTGCAGCAATGGATGATTTCCAATTAATTCCAATGATAAGCAAGTGTAGAACTAAGGAGGGAAGGCGAAAGACCAATTTGTACGGTTTCATCATAAAAGGAAGATCCCACTTAAGGAATGACACCGATGTGGTAAACTTTGTGAGCATGGAGTTTTCCCTCACTGACCCAAGACTTGAACCACACAAATGGGAGAAGTACTGTGTTCTTGAGGTAGGAGATATGCTTCTAAGAAGTGCCATAGGCCATGTGTCAAGGCCTATGTTCTTGTATGTGAGGACAAATGGAACCTCAAAAATTAAAATGAAATGGGGGATGGAAATGAGGCGTTGCCTCCTTCAGTCACTTCAACAAATCGAGAGTATGATTGAAGCTGAGTCCTCTGTCAAGGAGAAAGACATGACCAAAGAGTTCTTTGAAAACAAATCAGAAACATGGCCCGTTGGAGAGTCCCCCAAAGGAGTGGAGGAAGGTTCCATTGGGAAGGTCTGCAGAACTTTATTGGCAAAGTCGGTATTCAACAGCTTGTATGCATCTCCACAACTAGAAGGATTTTCAGCTGAATCAAGAAAACTGCTTCTTATCGTTCAGGCTCTTAGGGACAACCTGGAACCTGGGACCTTTGATCTTGGGGGGCTATATGAAGCAATTGAGGAGTGCCTGATTAATGATCCCTGGGTTTTGCTTAATGCTTCTTGGTTCAACTCCTTCCTCACACATGCATTGAGATAGTTGTGGCAATGCTACTATTTGCTATCCATACTGTCCAAAAAAGTACCTTGTTTCTACT

>WSN_WT_HA

ATGAAGGCAAAACTACTGGTCCTGTTATATGCATTTGTAGCTACAGATGCAGACACAATATGTATAGGCTACCATGCGAACAACTCAACCGACACTGTTGACACAATACTCGAGAAGAATGTGGCAGTGACACATTCTGTTAACCTGCTCGAAGACAGCCACAACGGGAAACTATGTAAATTAAAAGGAATAGCCCCACTACAATTGGGGAAATGTAACATCACCGGATGGCTCTTGGGAAATCCAGAATGCGACTCACTGCTTCCAGCGAGATCATGGTCCTACATTGTAGAAACACCAAACTCTGAGAATGGAGCATGTTATCCAGGAGATCTCATCGACTATGAGGAACTGAGGGAGCAATTGAGCTCAGTATCATCATTAGAAAGATTCGAAATATTTCCCAAGGAAAGTTCATGGCCCAACCACACATTCAACGGAGTAACAGTATCATGCTCCCATAGGGGAAAAAGCAGTTTTTACAGAAATTTGCTATGGCTGACGAAGAAGGGGGATTCATACCCAAAGCTGACCAATTCCTATGTGAACAATAAAGGGAAAGAAGTCCTTGTACTATGGGGTGTTCATCACCCGTCTAGCAGTGATGAGCAACAGAGTCTCTATAGTAATGGAAATGCTTATGTCTCTGTAGCGTCTTCAAATTATAACAGGAGATTCACCCCGGAAATAGCTGCAAGGCCCAAAGTAAGAGATCAACATGGGAGGATGAACTATTACTGGACCTTGCTAGAACCCGGAGACACAATAATATTTGAGGCAACTGGTAATCTAATAGCACCATGGTATGCTTTCGCACTGAGTAGAGGGTTTGAGTCCGGCATCATCACCTCAAACGCGTCAATGCATGAGTGTAACACGAAGTGTCAAACACCCCAGGGAGCTATAAACAGCAATCTCCCTTTCCAGAATATACACCCAGTCACAATAGGAGAGTGCCCAAAATATGTCAGGAGTACCAAATTGAGGATGGTTACAGGACTAAGAAACATCCCATCCATTCAATACAGAGGTCTATTTGGAGCCATTGCTGGTTTTATTGAGGGGGGATGGACTGGAATGATAGATGGATGGTATGGTTATCATCATCAGAATGAACAGGGATCAGGCTATGCAGCGGATCAAAAAAGCACACAAAATGCCATTAACGGGATTACAAACAAGGTGAACTCTGTTATCGAGAAAATGAACACTCAATTCACAGCTGTGGGTAAAGAATTCAACAACTTAGAAAAAAGGATGGAAAATTTAAATAAAAAAGTTGATGATGGGTTTCTGGACATTTGGACATATAATGCAGAATTGTTAGTTCTACTGGAAAATGAAAGGACTTTGGATTTCCATGACTTAAATGTGAAGAATCTGTACGAGAAAGTAAAAAGCCAATTAAAGAATAATGCCAAAGAAATCGGAAATGGGTGTTTTGAGTTCTACCACAAGTGTGACAATGAATGCATGGAAAGTGTAAGAAATGGGACTTATGATTATCCAAAATATTCAGAAGAATCAAAGTTGAACAGGGAAAAGATAGATGGAGTGAAATTGGAATCAATGGGGGTGTATCAGATTCTGGCGATCTACTCAACTGTCGCCAGTTCACTGGTGCTTTTGGTCTCCCTGGGGGCAATCAGTTTCTGGATGTGTTCTAATGGGTCTTTGCAGTGCAGAATATGCATCTGAGATTAGGATTTCAGAAATATAAGGAAAAACACCCTTGTTTCTACT

>WSN_WT_NP

ATGGCGACCAAAGGCACCAAACGATCTTACGAACAGATGGAGACTGATGGAGAACGCCAGAATGCCACTGAAATCAGAGCATCTGTCGGAAAAATGATTGATGGAATTGGACGATTCTACATCCAAATGTGCACCGAACTTAAACTCAGTGATTATGAGGGACGGCTGATTCAGAACAGCTTAACAATAGAGAGAATGGTGCTCTCTGCTTTTGACGAGAGGAGGAATAAATATCTAGAAGAACATCCCAGTGCGGGGAAAGATCCTAAGAAAACTGGAGGACCTATATACAGGAGAGTAGATGGAAAGTGGAGGAGAGAACTCATCCTTTATGACAAAGAAGAAATAAGACGAATCTGGCGCCAAGCTAATAATGGTGACGATGCAACGGCTGGTCTGACTCACATGATGATCTGGCACTCCAATTTGAATGATGCAACTTACCAGAGGACAAGAGCTCTTGTTCGCACAGGAATGGATCCCAGGATGTGCTCACTGATGCAGGGTTCAACCCTCCCTAGGAGGTCTGGGGCCGCAGGTGCTGCAGTCAAAGGAGTTGGAACAATGGTGATGGAATTGATCAGAATGATCAAACGTGGGATCAATGATCGGAACTTCTGGAGGGGTGAGAATGGACGGAGAACAAGGATTGCTTATGAAAGAATGTGCAACATTCTCAAAGGGAAATTTCAAACAGCTGCACAAAGAACAATGGTGGATCAAGTGAGAGAGAGCCGGAATCCAGGAAATGCTGAGTTCGAAGATCTCATCTTTTTAGCACGGTCTGCACTCATATTGAGAGGGTCAGTTGCTCACAAGTCCTGCCTGCCTGCCTGTGTGTATGGATCTGCCGTAGCCAGTGGATACGACTTTGAAAGAGAGGGATACTCTCTAGTCGGAATAGACCCTTTCAGACTGCTTCAAAACAGCCAAGTATACAGCCTAATCAGACCAAATGAGAATCCAGCACACAAGAGTCAACTGGTGTGGATGGCATGCCATTCTGCTGCATTTGAAGATCTAAGAGTATCAAGCTTCATCAGAGGGACGAAAGTGGTCCCAAGAGGGAAGCTTTCCACTAGAGGAGTTCAAATTGCTTCCAATGAAAACATGGAGACTATGGAATCAAGTACCCTTGAACTGAGAAGCAGATACTGGGCCATAAGGACCAGAAGTGGAGGGAACACCAATCAACAGAGGGCTTCCTCGGGCCAAATCAGCATACAACCTACGTTCTCAGTACAGAGAAATCTCCCTTTTGACAGACCAACCATTATGGCAGCATTCACTGGGAATACAGAGGGGAGAACATCTGACATGAGAACCGAAATCATAAGGCTGATGGAAAGTGCAAGACCAGAAGATGTGTCTTTCCAGGGGCGGGGAGTCTTCGAGCTCTCGGACGAAAAGGCAACGAGCCCGATCGTGCCCTCCTTTGACATGAGTAATGAAGGATCTTATTTCTTCGGAGACAATGCAGAGGAGTACGACAATTAAAGAAAAATACCCTTGTTTCTACT

>WSN_WT_NA

ATGAATCCAAACCAGAAAATAATAACCATTGGGTCAATCTGTATGGTAGTCGGAATAATTAGCCTAATATTGCAAATAGGAAATATAATCTCAATATGGATTAGCCATTCAATTCAAACCGGAAATCAAAACCATACTGGAATATGCAACCAAGGCAGCATTACCTATAAAGTTGTTGCTGGGCAGGACTCAACTTCAGTGATATTAACCGGCAATTCATCTCTTTGTCCCATCCGTGGGTGGGCTATACACAGCAAAGACAATGGCATAAGAATTGGTTCCAAAGGAGACGTTTTTGTCATAAGAGAGCCTTTTATTTCATGTTCTCACTTGGAATGCAGGACCTTTTTTCTGACTCAAGGCGCCTTACTGAATGACAAGCATTCAAGGGGGACCTTTAAGGACAGAAGCCCTTATAGGGCCTTAATGAGCTGCCCTGTCGGTGAAGCTCCGTCCCCGTACAATTCAAGGTTTGAATCGGTTGCTTGGTCAGCAAGTGCATGTCATGATGGAATGGGCTGGCTAACAATCGGAATTTCTGGTCCAGATGATGGAGCAGTGGCTGTATTAAAATACAACCGCATAATAACTGAAACCATAAAAAGTTGGAGGAAGAATATATTGAGAACACAAGAGTCTGAATGTACCTGTGTAAATGGTTCATGTTTTACCATAATGACCGATGGCCCAAGTGATGGGCTGGCCTCGTACAAAATTTTCAAGATCGAGAAGGGGAAGGTTACTAAATCGATAGAGTTGAATGCACCTAATTCTCACTACGAGGAATGTTCCTGTTACCCTGATACCGGCAAAGTGATGTGTGTGTGCAGAGACAATTGGCACGGTTCGAACCGACCATGGGTGTCCTTCGACCAAAACCTAGATTATAAAATAGGATACATCTGCAGTGGGGTTTTCGGTGACAACCCGCGTCCCAAAGATGGAACAGGCAGCTGTGGCCCAGTGTCTGCTGATGGAGCAAACGGAGTAAAGGGATTTTCATATAAGTATGGCAATGGTGTTTGGATAGGAAGGACTAAAAGTGACAGTTCCAGACATGGGTTTGAGATGATTTGGGATCCTAATGGATGGACAGAGACTGATAGTAGGTTCTCTATGAGACAAGATGTTGTGGCAATAACTAATCGGTCAGGGTACAGCGGAAGTTTCGTTCAACATCCTGAGCTAACAGGGCTAGACTGTATGAGGCCTTGCTTCTGGGTTGAATTAATCAGGGGGCTACCTGAGGAGGACGCAATCTGGACTAGTGGGAGCATCATTTCTTTTTGTGGTGTGAATAGTGATACTGTAGATTGGTCTTGGCCAGACGGTGCTGAGTTGCCGTTCACCATTGACAAGTAGTTTGTTCAAAAAACTCCTTGTTTCTACT

>WSN_WT_M

AGCAAAAGCAGGTAGATATTGAAAGATGAGTCTTCTAACCGAGGTCGAAACGTACGTTCTCTCTATCGTCCCGTCAGGCCCCCTCAAAGCCGAGATCGCACAGAGACTTGAAGATGTCTTTGCAGGGAAGAACACCGATCTTGAGGTTCTCATGGAATGGCTAAAGACAAGACCAATCCTGTCACCTCTGACTAAGGGGATTTTAGGATTTGTGTTCACGCTCACCGTGCCCAGTGAGCGGGGACTGCAGCGTAGACGCTTTGTCCAAAATGCTCTTAATGGGAACGGAGATCCAAATAACATGGACAAAGCAGTTAAACTGTATAGGAAGCTTAAGAGGGAGATAACATTCCATGGGGCCAAAGAAATAGCACTCAGTTATTCTGCTGGTGCACTTGCCTGTTGTATGGGCCTCATATACAACAGGATGGGGGCTGTGACCACTGAAGTGGCATTTGGCCTGGTATGCGCAACCTGTGAACAGATTGCTGACTCCCAGCATCGGTCTCATAGGCAAATGGTGACAACAACCAATCCACTAATCAGACATGAGAACAGAATGGTTCTAGCCAGCACTACAGCTAAGGCTATGGAGCAAATGGCTGGATCGAGTGAGCAAGCAGCAGAGGCCATGGATATTGCTAGTCAGGCCAGGCAAATGGTGCAGGCGATGAGAACCGTTGGGACTCATCCTAGCTCCAGTGCTGGTCTAAAAGATGATCTTCTTGAAAATTTACAGGCCTATCAGAAACGAATGGGGGTGCAGATGCAACGATTCAAGTGA

>WSN_WT_NS

AGCAAAAGCAGGGTGACAAAGACATAATGGATCCAAACACTGTGTCAAGCTTTCAGGTAGATTGCTTTCTTTGGCATGTCCGCAAAAGAGTTGCAGACCAAGAACTAGGTGATGCCCCATTCCTTGATCGGCTTCGCCGAGATCAGAAGTCCCTAAGAGGAAGAGGCAGCACTCTtGGTCTGGACATCGAAACAGCCACCCGTGCTGGAAAGCAAATAGTGGAGCGGATTCTGAAGGAAGAATCTGATGAGGCACTCAAAATGACCATGGCCTCTGTACCTGCATCGCGCTACCTAACTGACATGACTCTTGAGGAAATGTCAAGGCACTGGTTCATGCTCATGCCCAAGCAGAAAGTGGCAGGCCCTCTTTGTATCAGAATGGACCAGGCGATCATGGATAAGAACATCATACTGAAAGCGAACTTCAGTGTGATTTTTGACCGGCTGGAGACTCTAATATTACTAAGGGCCTTCACCGAAGAGGGGACAATTGTTGGCGAAATTTCACCACTGCCCTCTCTTCCAGGACATACTGATGAGGATGTCAAAAATGCAGTTGGGGTCCTCATCGGAGGACTTGAATGGAATAATAACACAGTTCGAGTCTCTGAAACTCTACAGAGATTCGCTTGGAGAAGCAGTAATGAGAATGGGAGACCTCCACTCACTCCAAAACAGAAACGGAAAATGGCGGGAACAATTAGGTCAGAAGTTTGA

**Fig S1 B: Nucleotide sequence of IAV CpG-high sequences given per segment**

**>CpG_high_PB2**

ATGGAAAGAATAAAAGAACTAAGGAATCTAATGTCGCAGTCTCGCACTCGCGAGATACTCACAAAAACCACCGTGGACCATATGGCCATAATCAAGAAGTACACATCAGGAAGACAGGAGAAGAACCCAGCACTTAGGATGAAATGGATGATGGCGATGAAATATCCGATTACAGCGGACAAGAGGATAACGGAAATGATTCCGGAGAGAAACGAGCAGGGACAAACGTTATGGAGTAAAATGAACGACGCCGGATCGGACCGAGTGATGGTATCACCTCTGGCCGTGACATGGTGGAATAGGAATGGACCCGTGACAAGTACGGTTCATTATCCAAAAATCTACAAAACTTATTTCGAAAAAGTCGAAAGGTTAAAACATGGAACCTTCGGCCCCGTCCATTTTCGAAACCAAGTCAAAATACGTCGACGAGTCGACATAAATCCTGGTCATGCAGATCTCAGCGCCAAAGAGGCACAGGATGTAATCATGGAAGTCGTTTTCCCTAACGAAGTGGGAGCCAGGATACTAACGTCGGAATCGCAACTAACGACAACGAAAGAGAAGAAAGAAGAACTCCAGGGTTGCAAAATTTCTCCTCTGATGGTGGCGTACATGTTGGAGAGAGAACTGGTCCGCAAAACGAGATTCCTCCCGGTGGCCGGCGGAACAAGCAGTGTGTACATCGAAGTGTTGCATTTGACCCAAGGAACGTGCTGGGAACAGATGTACACTCCGGGCGGGGAGGCGAGGAACGACGATGTTGATCAAAGCTTAATTATCGCGGCTAGAAACATAGTAAGAAGAGCCACGGTATCAGCGGATCCACTAGCATCTTTATTGGAGATGTGCCACAGCACGCAGATTGGTGGAATAAGGATGGTAAACATCCTTAGGCAGAACCCAACAGAAGAGCAAGCCGTGGATATTTGCAAGGCTGCAATGGGACTGAGAATTAGCTCGTCCTTCAGTTTTGGCGGATTCACATTTAAGAGAACAAGCGGATCATCCGTCAAGAGAGAGGAAGAGGTGCTTACGGGCAATCTTCAGACATTGAAGATAAGAGTGCATGAGGGATATGAAGAGTTCACAATGGTTGGGAGAAGAGCAACAGCTATACTCAGAAAAGCAACCAGGAGATTGATTCAGCTGATAGTGAGCGGGCGAGACGAACAGTCGATCGCCGAAGCGATAATCGTGGCCATGGTATTTTCACAAGAGGATTGTATGATAAAAGCAGTTCGAGGCGACCTGAATTTCGTCAATAGGGCGAATCAGCGATTGAATCCCATGCACCAACTTTTGAGACATTTTCAGAAGGACGCGAAGGTGCTCTTTCAAAATTGGGGAATCGAATCCATCGACAATGTGATGGGAATGATCGGGATATTGCCCGACATGACTCCAAGCACCGAGATGTCGATGAGAGGAGTGAGAATCAGCAAAATGGGCGTAGATGAGTATTCCAGCGCGGAGAAGATAGTGGTGAGCATTGACCGTTTTTTGAGAGTTCGGGACCAACGCGGGAACGTACTACTGTCTCCCGAGGAGATCAGCGAAACGCAGGGAACAGAGAAACTGACAATAACTTACTCGTCGTCGATGATGTGGGAGATTAACGGTCCTGAATCGGTGTTGGTCAATACCTATCAGTGGATCATCAGAAACTGGGAAACTGTTAAAATTCAGTGGTCCCAGAATCCTACGATGCTGTACAATAAAATGGAATTCGAGCCGTTTCAGTCTTTAGTTCCAAAGGCCGTTAGAGGCCAATACAGTGGGTTCGTGAGAACTCTGTTCCAACAAATGCGGGACGTGCTCGGGACATTTGATACCGCTCAGATAATAAAACTTCTTCCCTTCGCAGCCGCTCCACCGAAGCAAAGTAGAACGCAGTTCTCGTCGTTGACTATAAATGTGAGGGGATCAGGAATGAGAATACTCGTAAGGGGCAATTCTCCAGTATTCAACTACAACAAGACCACGAAACGACTCACCGTTCTCGGAAAGGACGCCGGCCCTTTAACTGAAGACCCAGACGAAGGCACAGCCGGAGTTGAGTCCGCCGTTCTGAGAGGATTCCTCATTCTCGGCAAAGAAGACAGGAGATATGGACCAGCATTAAGCATAAATGAACTGAGCAACCTTGCGAAAGGAGAGAAGGCTAATGTGCTAATTGGGCAAGGAGACGTGGTGTTGGTAATGAAACGGAAACGGAACTCTAGCATACTTACTGACAGCCAGACAGCGACCAAAAGAATTCGGATGGCCATCAATTAGTGTCGAATAGTTTAAAAACGACCTTGTTTCTACT

**>CpG_high_PB1**

ATGGATGTCAATCCGACTTTACTTTTCTTAAAAGTGCCAGCACAAAATGCTATAAGCACAACTTTCCCTTATACTGGAGACCCTCCTTACAGCCATGGGACAGGAACAGGATACACCATGGATACTGTCAACAGGACACATCAGTACTCAGAAAGGGGAAGATGGACAACAAACACCGAAACTGGAGCACCGCAACTCAACCCGATTGATGGGCCACTGCCAGAAGACAATGAACCAAGTGGTTATGCCCAAACAGATTGTGTATTGGAAGCAATGGCCTTCCTTGAGGAATCCCATCCTGGTATCTTTGAGACCTCGTGTCTTGAAACGATGGAGGTTGTTCAGCAAACACGAGTGGACAAGCTGACACAAGGCCGACAGACCTATGACTGGACTCTAAATAGGAACCAGCCGGCGGCAACGGCGTTGGCCAACACAATAGAAGTGTTCAGATCGAACGGCCTCACGGCCAATGAATCTGGACGGCTCATAGACTTCCTTAAGGATGTAATGGAGTCAATGAACAAAGAAGAAATGGAGATCACAACTCATTTTCAGAGAAAGAGACGAGTGAGAGACAATATGACTAAGAAAATGGTGACACAGAGAACAATAGGTAAAAGGAAGCAGAGATTGAACAAAAGGAGTTATCTAATTAGGGCATTAACCCTGAACACGATGACCAAAGATGCTGAGAGAGGGAAGCTAAAACGGCGAGCGATCGCAACCCCCGGGATGCAAATAAGGGGGTTCGTATACTTCGTCGAGACACTAGCGAGGAGTATATGCGAGAAACTTGAACAATCCGGATTGCCGGTCGGAGGCAACGAGAAGAAAGCAAAGTTGGCAAACGTCGTAAGGAAGATGATGACCAATTCTCAGGACACTGAAATTTCTTTCACCATCACCGGAGATAACACCAAATGGAACGAAAATCAGAACCCTCGGATGTTTTTGGCGATGATCACGTATATAACCAGAAATCAGCCCGAATGGTTCCGAAACGTTCTAAGTATCGCTCCAATAATGTTCTCAAACAAAATGGCGAGACTGGGAAAGGGGTACATGTTCGAGAGCAAGAGTATGAAAATTCGAACTCAAATACCCGCAGAAATGCTAGCAAGCATCGATTTGAAATACTTCAACGATTCAACGAGAAAGAAGATTGAAAAAATCCGGCCGCTCTTAATAGACGGGACTGCGTCATTGAGCCCTGGAATGATGATGGGCATGTTCAATATGTTAAGTACGGTATTAGGCGTCTCGATCCTGAATCTCGGACAAAAGAGACACACCAAGACTACTTACTGGTGGGACGGTCTTCAATCGTCCGACGATTTCGCTCTGATCGTGAACGCGCCGAATCATGAAGGGATTCAAGCCGGAGTCAACAGGTTTTATCGAACCTGTAAGCTACTCGGAATTAATATGAGCAAGAAAAAGTCTTACATAAACCGAACAGGTACATTTGAATTCACGAGTTTTTTCTATCGTTATGGGTTTGTTGCCAATTTCAGCATGGAGCTTCCCAGCTTTGGGGTGTCTGGGATCAACGAGTCGGCGGACATGAGTATTGGAGTTACTGTCATCAAAAACAATATGATAAACAACGATCTTGGTCCAGCAACCGCTCAAATGGCCCTTCAGCTGTTCATCAAAGATTACAGGTACACGTACCGGTGCCATCGAGGTGACACGCAAATACAAACGCGAAGATCATTCGAAATAAAGAAACTGTGGGAGCAAACCCATTCCAAAGCTGGACTGCTGGTCTCCGACGGAGGCCCAAATTTATACAACATTCGAAATCTCCACATTCCCGAAGTCTGCTTGAAATGGGAATTAATGGATGAGGATTACCAGGGGCGTTTATGCAACCCACTGAACCCGTTCGTCAACCATAAAGACATTGAATCAGTGAACAATGCAGTGATAATGCCGGCACACGGTCCGGCCAAAAACATGGAGTATGACGCCGTTGCAACAACGCACTCCTGGATCCCCAAAAGAAATCGATCCATCTTGAATACGAGCCAAAGAGGAATACTTGAAGATGAACAAATGTACCAAAAGTGCTGCAACTTATTCGAAAAATTCTTCCCCAGCAGTTCGTACCGAAGACCGGTCGGGATATCCAGTATGGTGGAGGCTATGGTTTCCAGAGCCCGAATTGACGCACGAATCGATTTCGAATCTGGAAGGATAAAGAAAGAGGAGTTCACTGAGATCATGAAGATCTGTTCCACCATTGAAGAGCTCAGACGGCAAAAATAGTGAATTTAGCTTGTCCTTCATGAAAAAATGCCTTGTTTCTACT

**>CpG_high_PA**

ATGGAAGATTTTGTGCGACAATGCTTCAATCCGATGATTGTCGAGCTTGCGGAAAAGGCAATGAAAGAGTATGGAGAGGACCTGAAAATCGAAACAAACAAATTTGCAGCAATATGCACTCACTTGGAAGTGTGCTTCATGTATTCAGATTTTCACTTCATCGATGAGCAAGGCGAGTCAATAGTCGTAGAACTTGGCGATCCAAATGCACTTTTGAAGCACAGATTTGAAATAATCGAGGGAAGAGATCGCACAATAGCCTGGACAGTAATAAACAGTATTTGCAACACTACAGGGGCCGAGAAACCGAAGTTTCTACCGGATTTGTACGATTACAAGAAGAATCGATTCATCGAAATTGGAGTAACACGGAGAGAAGTTCACATATACTATCTGGAAAAGGCCAATAAAATTAAATCCGAGAAGACACACATCCACATTTTCTCATTCACTGGGGAGGAAATGGCCACAAAGGCCGACTACACTCTCGACGAAGAAAGCAGGGCTAGGATCAAAACCAGGCTATTCACCATAAGACAAGAAATGGCTAGCAGAGGCCTCTGGGATTCCTTTCGTCAGTCCGAGAGAGGCGAAGAGACAATTGAAGAAAGATTTGAAATCACAGGAACAATGCGCAAGCTTGCCGACCAAAGTCTCCCGCCAAACTTCTCCAGCCTTGAAAAATTTAGAGCCTATGTGGATGGATTCGAACCGAACGGCTACATTGAGGGCAAGCTTTCTCAAATGTCCAAAGAAGTAAATGCTAGAATTGAACCTTTTTTGAAATCAACACCACGACCACTTAGACTTCCGGATGGGCCTCCCTGTTCTCAGCGGTCGAAATTCCTGCTGATGGATGCCTTAAAATTAAGCATCGAGGACCCGAGTCACGAGGGAGAGGGGATACCGCTATACGATGCGATCAAATGCATGAGAACATTCTTCGGATGGAAGGAACCCAATGTCGTTAAACCACACGAAAAGGGAATAAATCCGAATTATCTTCTGTCGTGGAAGCAAGTACTGGCAGAACTGCAGGACATTGAGAATGAGGAGAAAATTCCAAGGACTAAAAATATGAAGAAAACGAGTCAGTTAAAGTGGGCACTCGGTGAGAACATGGCACCAGAAAAGGTAGACTTTGACGATTGTAAAGATGTAGGCGATTTGAAGCAATATGATAGTGATGAACCAGAATTGAGGTCGCTTGCAAGTTGGATTCAGAATGAGTTCAACAAGGCATGTGAACTGACCGATTCAAGCTGGATAGAGCTCGATGAGATTGGAGAAGACGCGGCTCCGATCGAACACATCGCAAGCATGAGAAGGAATTATTTCACAGCGGAGGTGTCTCATTGCAGAGCCACAGAATACATAATGAAGGGGGTGTACATCAATACGGCCTTGCTTAATGCATCCTGTGCAGCAATGGACGATTTCCAATTAATTCCAATGATAAGCAAGTGTAGAACTAAGGAGGGAAGGCGAAAGACCAATTTGTACGGTTTCATCATAAAAGGAAGATCCCACTTAAGGAATGACACCGATGTGGTAAACTTTGTGAGCATGGAGTTTTCCCTCACTGACCCGAGACTTGAACCACACAAATGGGAGAAGTACTGTGTTCTTGAGGTAGGAGATATGCTTCTAAGAAGTGCCATAGGCCATGTGTCAAGGCCTATGTTCTTGTATGTGAGGACAAATGGAACCTCAAAAATTAAAATGAAATGGGGGATGGAAATGAGGCGTTGCCTCCTTCAGTCACTTCAACAAATCGAGAGTATGATTGAAGCTGAGTCCTCTGTCAAGGAGAAAGACATGACCAAAGAGTTCTTTGAAAACAAATCAGAAACATGGCCCGTCGGAGAGTCCCCCAAAGGAGTGGAGGAAGGTTCCATCGGGAAGGTCTGCAGAACTTTATTGGCGAAGTCGGTATTCAACAGCTTGTATGCATCTCCACAACTAGAAGGATTTTCAGCTGAATCAAGAAAACTGCTTCTTATCGTTCAGGCTCTTAGGGACAACCTGGAACCTGGGACCTTTGATCTTGGGGGGCTATATGAAGCAATTGAGGAGTGCCTGATTAATGATCCCTGGGTTTTGCTTAATGCTTCTTGGTTCAACTCCTTCCTCACACATGCATTGAGATAGTTGTGGCAATGCTACTATTTGCTATCCATACTGTCCAAAAAAGTACCTTGTTTCTACT

**>CpG_high_HA**

ATGAAGGCAAAACTACTGGTCCTGTTATATGCATTTGTAGCTACAGATGCAGACACAATATGTATCGGCTACCATGCGAACAACTCGACCGACACTGTTGACACAATACTCGAGAAGAACGTGGCAGTGACACATTCTGTTAACCTGCTCGAAGACAGCCACAACGGGAAACTATGTAAATTAAAAGGAATAGCCCCACTACAATTGGGGAAATGTAACATCACCGGATGGCTCTTGGGAAATCCAGAATGCGACTCACTGCTTCCAGCGAGATCATGGTCCTACATTGTAGAAACACCAAACTCCGAGAATGGAGCATGTTATCCAGGAGATCTCATCGACTATGAGGAACTGAGGGAGCAATTGAGCTCAGTATCATCGTTAGAAAGATTCGAAATATTTCCCAAGGAAAGTTCATGGCCCAACCACACGTTCAACGGAGTAACGGTATCATGCTCCCATAGGGGAAAAAGCAGTTTTTACAGAAATTTGCTATGGCTGACGAAGAAGGGGGATTCATACCCAAAGCTGACCAATTCCTATGTGAACAATAAAGGGAAAGAAGTCCTCGTACTATGGGGTGTTCATCACCCGTCTAGCAGTGATGAGCAACAGAGTCTCTATAGTAATGGAAATGCTTATGTCTCTGTAGCGTCTTCAAATTATAACAGGAGATTCACCCCGGAAATAGCTGCAAGGCCCAAAGTAAGAGATCAACATGGGAGGATGAACTATTACTGGACCTTGCTAGAACCCGGAGACACAATAATATTTGAGGCAACTGGTAATCTAATAGCACCATGGTATGCTTTCGCACTGAGTAGAGGGTTTGAGTCCGGCATCATCACCTCAAACGCGTCAATGCACGAGTGTAACACGAAGTGTCAAACACCCCAGGGAGCTATAAACAGCAATCTCCCTTTCCAGAATATACACCCAGTCACAATAGGAGAGTGCCCAAAATATGTCAGGAGTACCAAATTGAGGATGGTTACAGGACTAAGAAACATCCCATCCATTCAATACAGAGGTCTATTTGGAGCCATTGCTGGTTTTATTGAGGGGGGATGGACTGGAATGATAGATGGATGGTACGGTTATCATCATCAGAATGAACAGGGATCAGGCTATGCAGCGGATCAAAAAAGCACACAAAATGCCATTAACGGGATTACAAACAAGGTGAACTCTGTTATCGAGAAAATGAACACTCAATTCACAGCTGTGGGTAAAGAATTCAACAACTTAGAAAAAAGGATGGAAAATTTAAATAAAAAAGTTGATGACGGGTTTCTGGACATTTGGACATATAACGCCGAATTGTTAGTTCTACTGGAAAATGAAAGGACTTTGGATTTCCACGACTTAAACGTGAAGAATCTGTACGAGAAAGTAAAAAGCCAATTAAAGAATAATGCCAAAGAAATCGGAAACGGGTGTTTTGAGTTCTACCACAAGTGCGACAATGAATGCATGGAAAGTGTAAGAAATGGGACTTATGATTATCCAAAATATTCCGAAGAATCAAAGTTGAACAGGGAAAAGATAGATGGAGTGAAATTGGAATCAATGGGGGTGTATCAGATTCTGGCGATCTACTCAACTGTCGCCAGTTCACTGGTGCTTTTGGTCTCCCTGGGGGCAATCAGTTTCTGGATGTGTTCTAATGGGTCTTTGCAGTGCAGAATATGCATCTGAGATTAGGATTTCAGAAATATAAGGAAAAACACCCTTGTTTCTACT

**>CpG_high_NP**

ATGGCGACCAAAGGCACCAAACGATCTTACGAACAGATGGAGACTGATGGAGAACGCCAGAATGCCACTGAAATCAGAGCATCTGTCGGAAAAATGATTGATGGAATTGGACGATTCTACATCCAAATGTGCACCGAACTTAAACTCAGTGATTATGAGGGACGGCTGATTCAGAACAGCTTAACAATAGAGAGAATGGTGCTCTCTGCTTTCGACGAGAGGAGGAATAAATATCTAGAAGAACATCCCAGCGCGGGGAAAGATCCGAAGAAAACTGGAGGACCTATATACCGGAGAGTCGACGGAAAGTGGAGGAGAGAACTCATCCTTTACGACAAAGAAGAAATAAGACGAATCTGGCGCCAAGCGAATAATGGCGACGACGCGACGGCTGGTCTGACTCACATGATGATCTGGCACTCCAATTTGAATGATGCAACTTACCAGAGGACAAGAGCTCTTGTTCGCACAGGAATGGATCCCAGGATGTGCTCACTGATGCAGGGTTCAACCCTCCCTAGGAGGTCTGGGGCCGCAGGTGCTGCAGTCAAAGGAGTTGGAACAATGGTGATGGAATTGATCCGAATGATCAAACGTGGGATCAACGATCGGAACTTCTGGAGGGGCGAGAATGGACGGAGAACAAGGATTGCTTACGAAAGAATGTGCAACATTCTCAAAGGGAAATTTCAAACAGCTGCACAAAGAACAATGGTGGATCAAGTGCGAGAGAGCCGGAATCCAGGAAACGCTGAGTTCGAAGATCTCATCTTTTTAGCACGGTCTGCACTCATATTGAGAGGGTCAGTTGCTCACAAGTCCTGCCTGCCCGCCTGTGTGTACGGATCTGCCGTAGCCAGTGGATACGACTTCGAAAGAGAGGGATACTCTCTAGTCGGAATAGACCCTTTCAGACTGCTTCAAAACAGCCAAGTATACAGCCTAATCAGACCGAACGAGAATCCCGCACACAAGAGTCAACTGGTGTGGATGGCATGCCATTCCGCTGCATTTGAAGATCTAAGAGTATCAAGCTTCATCAGAGGGACGAAAGTGGTCCCAAGAGGGAAGCTTTCCACTAGAGGAGTTCAAATTGCTTCCAATGAAAACATGGAGACTATGGAATCAAGTACCCTTGAACTGAGAAGCAGATACTGGGCCATAAGGACCAGAAGTGGAGGGAACACCAATCAACAGAGGGCTTCCTCGGGCCAAATCAGCATACAACCTACGTTCTCAGTACAGAGAAATCTCCCTTTCGACCGACCGACCATTATGGCAGCATTCACCGGGAATACGGAGGGGCGAACGTCCGACATGCGAACCGAAATCATAAGGCTGATGGAAAGTGCAAGACCAGAAGATGTGTCGTTCCAGGGGCGGGGAGTCTTCGAGCTCTCGGACGAAAAGGCAACGAGCCCGATCGTGCCCTCCTTTGACATGAGTAATGAAGGATCTTATTTCTTCGGAGACAATGCAGAGGAGTACGACAATTAAAGAAAAATACCCTTGTTTCTACT

**>CpG_high_NA**

ATGAATCCAAACCAGAAAATAATAACCATTGGGTCAATCTGTATGGTAGTCGGAATAATTAGCCTAATATTGCAAATAGGAAATATAATCTCGATATGGATTAGCCATTCAATTCAAACCGGAAATCAAAACCATACTGGAATATGCAACCAAGGCAGCATTACCTATAAAGTTGTTGCTGGGCAGGACTCAACGTCCGTGATATTAACCGGCAATTCATCTCTTTGTCCCATCCGCGGGTGGGCTATACACAGCAAAGACAATGGCATAAGAATTGGTTCCAAAGGAGACGTTTTTGTCATAAGAGAGCCGTTTATTTCATGTTCTCACTTGGAATGCAGGACCTTTTTTCTGACTCAAGGCGCCTTACTGAATGACAAGCATTCAAGGGGGACCTTTAAGGACCGAAGCCCTTATCGGGCCTTAATGAGCTGCCCTGTCGGTGAAGCTCCGTCCCCGTACAATTCAAGGTTTGAATCGGTTGCTTGGTCAGCAAGTGCATGTCATGATGGAATGGGCTGGCTAACAATCGGAATTTCTGGTCCAGATGATGGAGCAGTGGCTGTATTAAAATACAACCGCATAATAACTGAAACCATAAAAAGTTGGAGGAAGAATATATTGAGAACACAAGAGTCTGAATGTACCTGTGTAAACGGTTCATGTTTTACCATAATGACCGATGGCCCGAGTGATGGGCTCGCCTCGTACAAAATTTTCAAGATCGAGAAGGGGAAGGTTACTAAATCGATCGAGTTGAATGCACCTAATTCTCACTACGAGGAATGTTCCTGTTACCCTGATACCGGCAAAGTGATGTGTGTGTGCAGAGACAATTGGCACGGTTCGAACCGACCGTGGGTGTCCTTCGACCAAAACCTAGATTATAAAATAGGATACATCTGCAGTGGGGTTTTCGGTGACAACCCGCGTCCCAAAGATGGAACAGGCAGCTGTGGCCCGGTGTCGGCTGACGGAGCAAACGGAGTAAAGGGATTTTCATATAAGTACGGCAATGGTGTTTGGATCGGAAGGACTAAAAGTGACAGTTCCAGACACGGGTTTGAGATGATTTGGGATCCGAACGGATGGACAGAGACCGATAGTAGGTTCTCGATGAGACAAGATGTCGTGGCAATAACTAATCGGTCAGGGTACAGCGGAAGTTTCGTTCAACATCCTGAGCTAACAGGGCTAGACTGTATGAGGCCTTGCTTCTGGGTTGAATTAATCAGGGGGCTACCTGAGGAGGACGCAATCTGGACTAGTGGGAGCATCATTTCTTTTTGTGGTGTGAATAGTGATACTGTAGATTGGTCTTGGCCAGACGGTGCTGAGTTGCCGTTCACCATTGACAAGTAGTTTGTTCAAAAAACTCCTTGTTTCTACT

**Fig S1C: Nucleotide sequence of IAV CpG-low sequences given per segment**

**>CpG_low_PB2**

ATGGAAAGAATAAAAGAACTAAGGAATCTAATGTCGCAGTCTCGCACTCGCGAGATACTCACAAAAACCACCGTGGACCATATGGCCATAATCAAGAAGTACACATCAGGAAGACAGGAGAAGAACCCAGCACTTAGGATGAAATGGATGATGGCAATGAAATATCCAATTACAGCAGACAAGAGGATAACAGAAATGATTCCTGAGAGAAATGAGCAGGGACAAACTTTATGGAGTAAAATGAATGATGCTGGATCAGATAGAGTGATGGTATCACCTCTGGCTGTGACATGGTGGAATAGGAATGGACCAGTGACAAGTACAGTTCATTATCCAAAAATCTACAAAACTTATTTTGAAAAAGTTGAAAGGTTAAAACATGGAACCTTTGGCCCTGTCCATTTTAGAAACCAAGTCAAAATAAGGAGGAGAGTTGACATAAATCCTGGTCATGCAGATCTCAGTGCCAAAGAGGCACAGGATGTAATCATGGAAGTTGTTTTCCCTAATGAAGTGGGAGCCAGGATACTAACATCAGAATCACAACTAACAACAACCAAAGAGAAGAAAGAAGAACTCCAGGGTTGCAAAATTTCTCCTCTGATGGTGGCATACATGTTGGAGAGAGAACTGGTCCGCAAAACAAGATTCCTCCCAGTGGCTGGTGGAACAAGCAGTGTGTACATTGAAGTGTTGCATTTGACCCAAGGAACATGCTGGGAACAGATGTACACTCCAGGAGGGGAGGCGAGGAATGATGATGTTGATCAAAGCTTAATTATTGCTGCTAGAAACATAGTAAGAAGAGCCACAGTATCAGCAGATCCACTAGCATCTTTATTGGAGATGTGCCACAGCACGCAGATTGGTGGAATAAGGATGGTAAACATCCTTAGGCAGAACCCAACAGAAGAGCAAGCCGTGGATATTTGCAAGGCTGCAATGGGACTGAGAATTAGCTCATCCTTCAGTTTTGGTGGATTCACATTTAAGAGAACAAGTGGATCATCAGTCAAGAGAGAGGAAGAGGTGCTTACGGGCAATCTTCAGACATTGAAGATAAGAGTGCATGAGGGATATGAAGAGTTCACAATGGTTGGGAGAAGAGCAACAGCTATACTCAGAAAAGCAACCAGGAGATTGATTCAGCTGATAGTGAGTGGGAGAGATGAACAGTCAATTGCTGAAGCAATAATTGTGGCCATGGTATTTTCACAAGAGGATTGTATGATAAAAGCAGTTAGAGGTGACCTGAATTTTGTCAATAGGGCAAATCAGAGATTGAATCCCATGCACCAACTTTTGAGACATTTTCAGAAGGATGCAAAGGTGCTCTTTCAAAATTGGGGAATTGAATCCATTGACAATGTGATGGGAATGATTGGGATATTGCCAGACATGACTCCAAGCACAGAGATGTCAATGAGAGGAGTGAGAATCAGCAAAATGGGGGTAGATGAGTATTCCAGCGCGGAGAAGATAGTGGTGAGCATTGACCGTTTTTTGAGAGTTAGGGACCAAAGAGGGAATGTACTACTGTCTCCTGAGGAGATCAGTGAAACACAGGGAACAGAGAAACTGACAATAACTTACTCATCATCAATGATGTGGGAGATTAATGGTCCTGAATCAGTGTTGGTCAATACCTATCAGTGGATCATCAGAAACTGGGAAACTGTTAAAATTCAGTGGTCCCAGAATCCTACAATGCTGTACAATAAAATGGAATTTGAGCCATTTCAGTCTTTAGTTCCAAAGGCAGTTAGAGGCCAATACAGTGGGTTTGTGAGAACTCTGTTCCAACAAATGAGGGATGTGCTTGGGACATTTGATACTGCTCAGATAATAAAACTTCTTCCCTTTGCAGCTGCTCCACCAAAGCAAAGTAGAACGCAGTTCTCCTCATTGACTATAAATGTGAGGGGATCAGGAATGAGAATACTTGTAAGGGGCAATTCTCCAGTATTCAACTACAACAAGACCACTAAAAGACTCACAGTTCTTGGAAAGGATGCTGGCCCTTTAACTGAAGACCCAGATGAAGGCACAGCTGGAGTTGAGTCTGCAGTTCTGAGAGGATTCCTCATTCTGGGCAAAGAAGACAGGAGATATGGACCAGCATTAAGCATAAATGAACTGAGCAACCTTGCGAAAGGAGAGAAGGCTAATGTGCTAATTGGGCAAGGAGACGTGGTGTTGGTAATGAAACGGAAACGGAACTCTAGCATACTTACTGACAGCCAGACAGCGACCAAAAGAATTCGGATGGCCATCAATTAGTGTCGAATAGTTTAAAAACGACCTTGTTTCTACT

**>CpG_low_PB1**

ATGGATGTCAATCCGACTTTACTTTTCTTAAAAGTGCCAGCACAAAATGCTATAAGCACAACTTTCCCTTATACTGGAGACCCTCCTTACAGCCATGGGACAGGAACAGGATACACCATGGATACTGTCAACAGGACACATCAGTACTCAGAAAGGGGAAGATGGACAACAAACACCGAAACTGGAGCACCGCAACTCAACCCGATTGATGGGCCACTGCCAGAAGACAATGAACCAAGTGGTTATGCCCAAACAGATTGTGTATTGGAAGCAATGGCCTTCCTTGAGGAATCCCATCCTGGTATCTTTGAGACCTCGTGTCTTGAAACGATGGAGGTTGTTCAGCAAACACGAGTGGACAAGCTGACACAAGGCAGACAGACCTATGACTGGACTCTAAATAGGAACCAGCCTGCTGCAACAGCATTGGCCAACACAATAGAAGTGTTCAGATCAAATGGCCTCACGGCCAATGAATCTGGAAGGCTCATAGACTTCCTTAAGGATGTAATGGAGTCAATGAACAAAGAAGAAATGGAGATCACAACTCATTTTCAGAGAAAGAGACGAGTGAGAGACAATATGACTAAGAAAATGGTGACACAGAGAACAATAGGTAAAAGGAAGCAGAGATTGAACAAAAGGAGTTATCTAATTAGGGCATTAACCCTGAACACAATGACCAAAGATGCTGAGAGAGGGAAGCTAAAAAGAAGAGCAATTGCAACCCCAGGGATGCAAATAAGGGGGTTTGTATACTTTGTTGAGACACTAGCAAGGAGTATATGTGAGAAACTTGAACAATCAGGATTGCCAGTTGGAGGCAATGAGAAGAAAGCAAAGTTGGCAAATGTTGTAAGGAAGATGATGACCAATTCTCAGGACACTGAAATTTCTTTCACCATCACTGGAGATAACACCAAATGGAATGAAAATCAGAACCCTAGAATGTTTTTGGCCATGATCACATATATAACCAGAAATCAGCCTGAATGGTTCAGAAATGTTCTAAGTATTGCTCCAATAATGTTCTCAAACAAAATGGCAAGACTGGGAAAGGGGTACATGTTTGAGAGCAAGAGTATGAAAATTAGAACTCAAATACCTGCAGAAATGCTAGCAAGCATTGATTTGAAATACTTCAATGATTCAACTAGAAAGAAGATTGAAAAAATAAGGCCACTCTTAATAGATGGGACTGCATCATTGAGCCCTGGAATGATGATGGGCATGTTCAATATGTTAAGTACTGTATTAGGAGTCTCCATCCTGAATCTTGGACAAAAGAGACACACCAAGACTACTTACTGGTGGGATGGTCTTCAATCTTCTGATGATTTTGCTCTGATTGTGAATGCACCCAATCATGAAGGGATTCAAGCAGGAGTCAACAGGTTTTACAGGACCTGTAAGCTACTTGGAATTAATATGAGCAAGAAAAAGTCTTACATAAACAGAACAGGTACATTTGAATTCACAAGTTTTTTCTATCGTTATGGGTTTGTTGCCAATTTCAGCATGGAGCTTCCCAGCTTTGGGGTGTCTGGGATCAATGAGTCTGCTGACATGAGTATTGGAGTTACTGTCATCAAAAACAATATGATAAACAATGATCTTGGTCCAGCAACAGCTCAAATGGCCCTTCAGCTGTTCATCAAAGATTACAGGTACACATATAGGTGCCATAGAGGTGACACACAAATACAAACCCGAAGATCATTTGAAATAAAGAAACTGTGGGAGCAAACCCATTCCAAAGCTGGACTGCTGGTCTCAGATGGAGGCCCAAATTTATACAACATTAGAAATCTCCACATTCCTGAAGTCTGCTTGAAATGGGAATTAATGGATGAGGATTACCAGGGAAGACTATGCAACCCACTGAACCCATTTGTCAACCATAAAGACATTGAATCAGTGAACAATGCAGTGATAATGCCAGCACATGGTCCAGCCAAAAACATGGAGTATGATGCTGTTGCAACAACACACTCCTGGATCCCCAAAAGAAATCGATCCATCTTGAATACAAGCCAAAGAGGAATACTTGAAGATGAACAAATGTACCAAAAGTGCTGCAACTTATTTGAAAAATTCTTCCCCAGCAGTTCATACAGAAGACCAGTTGGGATATCCAGTATGGTGGAGGCTATGGTTTCCAGAGCCCGAATTGATGCACGAATTGATTTCGAATCTGGAAGGATAAAGAAAGAGGAGTTCACTGAGATCATGAAGATCTGTTCCACCATTGAAGAGCTCAGACGGCAAAAATAGTGAATTTAGCTTGTCCTTCATGAAAAAATGCCTTGTTTCTACT

**>CpG_low_PA**

ATGGAAGATTTTGTGCGACAATGCTTCAATCCGATGATTGTCGAGCTTGCGGAAAAGGCAATGAAAGAGTATGGAGAGGACCTGAAAATCGAAACAAACAAATTTGCAGCAATATGCACTCACTTGGAAGTGTGCTTCATGTATTCAGATTTTCACTTCATCGATGAGCAAGGCGAGTCAATAGTCGTAGAACTTGGCGATCCAAATGCACTTTTGAAGCACAGATTTGAAATAATCGAGGGAAGAGATCGCACAATAGCCTGGACAGTAATAAACAGTATTTGCAACACTACAGGGGCTGAGAAACCAAAGTTTCTACCAGATTTGTATGATTACAAGAAGAATAGATTCATTGAAATTGGAGTAACAAGGAGAGAAGTTCACATATACTATCTGGAAAAGGCCAATAAAATTAAATCTGAGAAGACACACATCCACATTTTCTCATTCACTGGGGAGGAAATGGCCACAAAGGCTGACTACACTCTTGATGAAGAAAGCAGGGCTAGGATCAAAACCAGGCTATTCACCATAAGACAAGAAATGGCTAGCAGAGGCCTCTGGGATTCCTTTCGTCAGTCCGAGAGAGGCGAAGAGACAATTGAAGAAAGATTTGAAATCACAGGAACAATGCGCAAGCTTGCCGACCAAAGTCTCCCGCCAAACTTCTCCAGCCTTGAAAAATTTAGAGCCTATGTGGATGGATTCGAACCGAACGGCTACATTGAGGGCAAGCTTTCTCAAATGTCCAAAGAAGTAAATGCTAGAATTGAACCTTTTTTGAAATCAACACCAAGACCACTTAGACTTCCTGATGGGCCTCCCTGTTCTCAGAGGTCCAAATTCCTGCTGATGGATGCCTTAAAATTAAGCATTGAGGACCCAAGTCATGAGGGAGAGGGGATACCTCTATATGATGCAATCAAATGCATGAGAACATTCTTTGGATGGAAGGAACCCAATGTTGTTAAACCACATGAAAAGGGAATAAATCCAAATTATCTTCTGTCATGGAAGCAAGTACTGGCAGAACTGCAGGACATTGAGAATGAGGAGAAAATTCCAAGGACTAAAAATATGAAGAAAACAAGTCAGTTAAAGTGGGCACTTGGTGAGAACATGGCACCAGAAAAGGTAGACTTTGACGATTGTAAAGATGTAGGCGATTTGAAGCAATATGATAGTGATGAACCAGAATTGAGGTCGCTTGCAAGTTGGATTCAGAATGAGTTCAACAAGGCATGTGAACTGACCGATTCAAGCTGGATAGAGCTTGATGAGATTGGAGAAGATGCAGCTCCAATTGAACACATTGCAAGCATGAGAAGGAATTATTTCACAGCAGAGGTGTCTCATTGCAGAGCCACAGAATACATAATGAAGGGGGTGTACATCAATACTGCCTTGCTTAATGCATCCTGTGCAGCAATGGATGATTTCCAATTAATTCCAATGATAAGCAAGTGTAGAACTAAGGAGGGAAGGCGAAAGACCAATTTGTACGGTTTCATCATAAAAGGAAGATCCCACTTAAGGAATGACACTGATGTGGTAAACTTTGTGAGCATGGAGTTTTCCCTCACTGACCCAAGACTTGAACCACACAAATGGGAGAAGTACTGTGTTCTTGAGGTAGGAGATATGCTTCTAAGAAGTGCCATAGGCCATGTGTCAAGGCCTATGTTCTTGTATGTGAGGACAAATGGAACCTCAAAAATTAAAATGAAATGGGGGATGGAAATGAGGCGTTGCCTCCTTCAGTCACTTCAACAAATCGAGAGTATGATTGAAGCTGAGTCCTCTGTCAAGGAGAAAGACATGACCAAAGAGTTCTTTGAAAACAAATCAGAAACATGGCCAGTTGGAGAGTCCCCCAAAGGAGTGGAGGAAGGTTCCATTGGGAAGGTCTGCAGAACTTTATTGGCAAAGTCGGTATTCAACAGCTTGTATGCATCTCCACAACTAGAAGGATTTTCAGCTGAATCAAGAAAACTGCTTCTTATCGTTCAGGCTCTTAGGGACAACCTGGAACCTGGGACCTTTGATCTTGGGGGGCTATATGAAGCAATTGAGGAGTGCCTGATTAATGATCCCTGGGTTTTGCTTAATGCTTCTTGGTTCAACTCCTTCCTCACACATGCATTGAGATAGTTGTGGCAATGCTACTATTTGCTATCCATACTGTCCAAAAAAGTACCTTGTTTCTACT

**>CpG_low_HA**

ATGAAGGCAAAACTACTGGTCCTGTTATATGCATTTGTAGCTACAGATGCAGACACAATATGTATAGGCTACCATGCTAACAACTCAACAGACACTGTTGACACAATACTAGAGAAGAATGTGGCAGTGACACATTCTGTTAACCTGCTAGAAGACAGCCACAATGGGAAACTATGTAAATTAAAAGGAATAGCCCCACTACAATTGGGGAAATGTAACATCACCGGATGGCTCTTGGGAAATCCAGAATGTGACTCACTGCTTCCAGCAAGATCATGGTCCTACATTGTAGAAACACCAAACTCTGAGAATGGAGCATGTTATCCAGGAGATCTCATCGACTATGAGGAACTGAGGGAGCAATTGAGCTCAGTATCATCATTAGAAAGATTTGAAATATTTCCCAAGGAAAGTTCATGGCCCAACCACACATTCAACGGAGTAACAGTATCATGCTCCCATAGGGGAAAAAGCAGTTTTTACAGAAATTTGCTATGGCTGACGAAGAAGGGGGATTCATACCCAAAGCTGACCAATTCCTATGTGAACAATAAAGGGAAAGAAGTCCTTGTACTATGGGGTGTTCATCACCCATCTAGCAGTGATGAGCAACAGAGTCTCTATAGTAATGGAAATGCTTATGTCTCTGTAGCGTCTTCAAATTATAACAGGAGATTCACCCCAGAAATAGCTGCAAGGCCCAAAGTAAGAGATCAACATGGGAGGATGAACTATTACTGGACCTTGCTAGAACCCGGAGACACAATAATATTTGAGGCAACTGGTAATCTAATAGCACCATGGTATGCTTTTGCACTGAGTAGAGGGTTTGAGTCTGGCATCATCACCTCAAACGCGTCAATGCATGAGTGTAACACGAAGTGTCAAACACCCCAGGGAGCTATAAACAGCAATCTCCCTTTCCAGAATATACACCCAGTCACAATAGGAGAGTGCCCAAAATATGTCAGGAGTACCAAATTGAGGATGGTTACAGGACTAAGAAACATCCCATCCATTCAATACAGAGGTCTATTTGGAGCCATTGCTGGTTTTATTGAGGGGGGATGGACTGGAATGATAGATGGATGGTATGGTTATCATCATCAGAATGAACAGGGATCAGGCTATGCAGCAGATCAAAAAAGCACACAAAATGCCATTAATGGGATTACAAACAAGGTGAACTCTGTTATTGAGAAAATGAACACTCAATTCACAGCTGTGGGTAAAGAATTCAACAACTTAGAAAAAAGGATGGAAAATTTAAATAAAAAAGTTGATGATGGGTTTCTGGACATTTGGACATATAATGCAGAATTGTTAGTTCTACTGGAAAATGAAAGGACTTTGGATTTCCATGACTTAAATGTGAAGAATCTGTATGAGAAAGTAAAAAGCCAATTAAAGAATAATGCCAAAGAAATTGGAAATGGGTGTTTTGAGTTCTACCACAAGTGTGACAATGAATGCATGGAAAGTGTAAGAAATGGGACTTATGATTATCCAAAATATTCAGAAGAATCAAAGTTGAACAGGGAAAAGATAGATGGAGTGAAATTGGAATCAATGGGGGTGTATCAGATTCTGGCGATCTACTCAACTGTCGCCAGTTCACTGGTGCTTTTGGTCTCCCTGGGGGCAATCAGTTTCTGGATGTGTTCTAATGGGTCTTTGCAGTGCAGAATATGCATCTGAGATTAGGATTTCAGAAATATAAGGAAAAACACCCTTGTTTCTACT

**>CpG_low_NP**

ATGGCGACCAAAGGCACCAAACGATCTTACGAACAGATGGAGACTGATGGAGAACGCCAGAATGCCACTGAAATCAGAGCATCTGTCGGAAAAATGATTGATGGAATTGGACGATTCTACATCCAAATGTGCACAGAACTTAAACTCAGTGATTATGAGGGAAGGCTGATTCAGAACAGCTTAACAATAGAGAGAATGGTGCTCTCTGCTTTTGATGAGAGGAGGAATAAATATCTAGAAGAACATCCCAGTGCAGGGAAAGATCCTAAGAAAACTGGAGGACCTATATACAGGAGAGTAGATGGAAAGTGGAGGAGAGAACTCATCCTTTATGACAAAGAAGAAATAAGAAGAATCTGGAGACAAGCTAATAATGGTGATGATGCAACAGCTGGTCTGACTCACATGATGATCTGGCACTCCAATTTGAATGATGCAACTTACCAGAGGACAAGAGCTCTTGTTCGCACAGGAATGGATCCCAGGATGTGCTCACTGATGCAGGGTTCAACCCTCCCTAGGAGGTCTGGGGCTGCAGGTGCTGCAGTCAAAGGAGTTGGAACAATGGTGATGGAATTGATCAGAATGATCAAAAGAGGGATCAATGATAGGAACTTCTGGAGGGGTGAGAATGGAAGGAGAACAAGGATTGCTTATGAAAGAATGTGCAACATTCTCAAAGGGAAATTTCAAACAGCTGCACAAAGAACAATGGTGGATCAAGTGAGAGAGAGCAGGAATCCAGGAAATGCTGAGTTTGAAGATCTCATCTTTTTAGCAAGGTCTGCACTCATATTGAGAGGGTCAGTTGCTCACAAGTCCTGCCTGCCTGCCTGTGTGTATGGATCTGCAGTAGCCAGTGGATATGACTTTGAAAGAGAGGGATACTCTCTAGTAGGAATAGACCCTTTCAGACTGCTTCAAAACAGCCAAGTATACAGCCTAATCAGACCAAATGAGAATCCAGCACACAAGAGTCAACTGGTGTGGATGGCATGCCATTCTGCTGCATTTGAAGATCTAAGAGTATCAAGCTTCATCAGAGGGACGAAAGTGGTCCCAAGAGGGAAGCTTTCCACTAGAGGAGTTCAAATTGCTTCCAATGAAAACATGGAGACTATGGAATCAAGTACCCTTGAACTGAGAAGCAGATACTGGGCCATAAGGACCAGAAGTGGAGGGAACACCAATCAACAGAGGGCTTCCTCAGGCCAAATCAGCATACAACCTACATTCTCAGTACAGAGAAATCTCCCTTTTGACAGACCAACCATTATGGCAGCATTCACTGGGAATACAGAGGGGAGAACATCTGACATGAGAACAGAAATCATAAGGCTGATGGAAAGTGCAAGACCAGAAGATGTGTCTTTCCAGGGGCGGGGAGTCTTCGAGCTCTCGGACGAAAAGGCAACGAGCCCGATCGTGCCCTCCTTTGACATGAGTAATGAAGGATCTTATTTCTTCGGAGACAATGCAGAGGAGTACGACAATTAAAGAAAAATACCCTTGTTTCTACT

**>CpG_low_NA**

ATGAATCCAAACCAGAAAATAATAACCATTGGGTCAATCTGTATGGTAGTAGGAATAATTAGCCTAATATTGCAAATAGGAAATATAATCTCAATATGGATTAGCCATTCAATTCAAACCGGAAATCAAAACCATACTGGAATATGCAACCAAGGCAGCATTACCTATAAAGTTGTTGCTGGGCAGGACTCAACTTCAGTGATATTAACAGGCAATTCATCTCTTTGTCCCATCAGAGGGTGGGCTATACACAGCAAAGACAATGGCATAAGAATTGGTTCCAAAGGAGATGTTTTTGTCATAAGAGAGCCTTTTATTTCATGTTCTCACTTGGAATGCAGGACCTTTTTTCTGACTCAAGGAGCCTTACTGAATGACAAGCATTCAAGGGGGACCTTTAAGGACAGAAGCCCTTATAGGGCCTTAATGAGCTGCCCTGTCGGTGAAGCTCCGTCCCCGTACAATTCAAGGTTTGAATCGGTTGCTTGGTCAGCAAGTGCATGTCATGATGGAATGGGCTGGCTAACAATCGGAATTTCTGGTCCAGATGATGGAGCAGTGGCTGTATTAAAATACAACAGAATAATAACTGAAACCATAAAAAGTTGGAGGAAGAATATATTGAGAACACAAGAGTCTGAATGTACCTGTGTAAATGGTTCATGTTTTACCATAATGACAGATGGCCCAAGTGATGGGCTGGCCTCCTACAAAATTTTCAAGATTGAGAAGGGGAAGGTTACTAAATCAATAGAGTTGAATGCACCTAATTCTCACTATGAGGAATGTTCCTGTTACCCTGATACAGGCAAAGTGATGTGTGTGTGCAGAGACAATTGGCATGGTTCAAACAGACCATGGGTGTCCTTTGACCAAAACCTAGATTATAAAATAGGATACATCTGCAGTGGGGTTTTTGGTGACAACCCAAGACCCAAAGATGGAACAGGCAGCTGTGGCCCAGTGTCTGCTGATGGAGCAAATGGAGTAAAGGGATTTTCATATAAGTATGGCAATGGTGTTTGGATAGGAAGGACTAAAAGTGACAGTTCCAGACATGGGTTTGAGATGATTTGGGATCCTAATGGATGGACAGAGACTGATAGTAGGTTCTCTATGAGACAAGATGTTGTGGCAATAACTAATAGGTCAGGGTACAGCGGAAGTTTCGTTCAACATCCTGAGCTAACAGGGCTAGACTGTATGAGGCCTTGCTTCTGGGTTGAATTAATCAGGGGGCTACCTGAGGAGGACGCAATCTGGACTAGTGGGAGCATCATTTCTTTTTGTGGTGTGAATAGTGATACTGTAGATTGGTCTTGGCCAGACGGTGCTGAGTTGCCGTTCACCATTGACAAGTAGTTTGTTCAAAAAACTCCTTGTTTCTACT
