# Supplementary material for: Increased or decreased numbers of CpG dinucleotide motifs in the genome of influenza A virus do not affect in vitro virus phenotype
Source: J Virol. 2026 Jun 22;100(7):e00047-26. doi: 10.1128/jvi.00047-26 (PMC13386996; doi:10.1128/jvi.00047-26)
Supplement: Fig. S2 — Mono-and di-nucleotide frequencies of WT and mutant viruses. [file jvi.00047-26-s0002.docx]

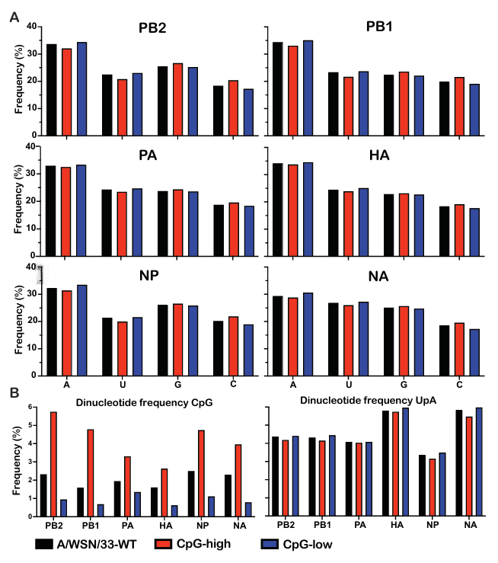


**Fig. S2 Mono- and di-nucleotide frequency analysis of six gene segments (PB2, PB1, PA, HA, NP and NA) of IAV mutants in which CpG dinucleotides were modified**. (A) Relative mononucleotide frequency was calculated by dividing the number of each nucleotide (A/U/G/C) to the total number of nucleotides in that particular segment. (B) Relative dinucleotide frequency of CpG/UpA was calculated by dividing the number of each dinucleotide to the total number of nucleotides present in that gene segment.
