# Supplementary figures and images for: Increased or decreased numbers of CpG dinucleotide motifs in the genome of influenza A virus do not affect in vitro virus phenotype
Source: J Virol. 2026 Jun 22;100(7):e00047-26. doi: 10.1128/jvi.00047-26 (PMC13386996; doi:10.1128/jvi.00047-26)

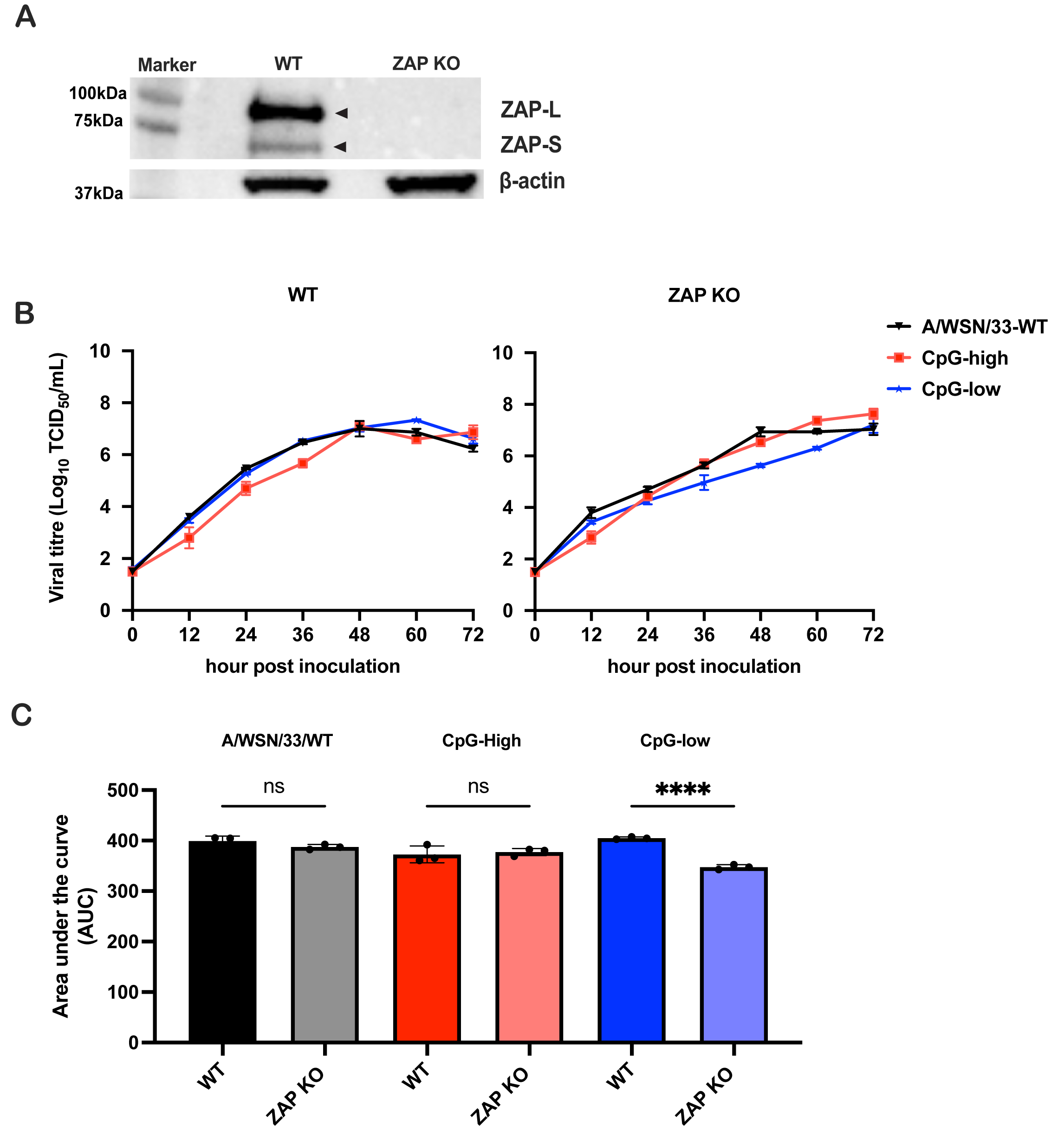

Supplement: Fig. S5 — Role of ZAP in recognition of IAV CpG mutants. [file jvi.00047-26-s0005.tif]
