# Supplementary material for: Increased or decreased numbers of CpG dinucleotide motifs in the genome of influenza A virus do not affect in vitro virus phenotype
Source: J Virol. 2026 Jun 22;100(7):e00047-26. doi: 10.1128/jvi.00047-26 (PMC13386996; doi:10.1128/jvi.00047-26)
Supplement: Supplemental legends — Descriptive legends for supplemental material. [file jvi.00047-26-s0006.docx]

**Captions Supplemental Materials**

**Fig S1:** Nucleotide sequence of A) A/WSN/33-WT, B) CpG-High and C) CpG-low,

sequences given per segment

**Fig. S2:** Mono- and di-nucleotide frequency analysis of six gene segments (PB2, PB1, PA, HA, NP and NA) of IAV mutants in which CpG dinucleotides were modified.

**Fig. S3:**. Table of SNPs with >1% mutation frequency detected in passaged viral genomes, stratified by nucleotide position, virus, duplicate, and passage.

**Fig. S4** Frequency of mono-and dinucleotide motif frequencies.

SNP analysis with a 1% variant frequency threshold was applied to virus genome sequences from P0 and P10. Positions exceeding this threshold and only present in P10, were used to quantify potential changes to alternative dinucleotide motifs for all three codon positions (1-2, 2-3, 3-1).

**Fig. S5: Role of Zinc-finger Antiviral Protein (ZAP) in recognition of IAV CpG mutants**. A) Protein expression of ZAP-L and ZAP-S isoforms in A549wt and ZAP KO cells, with β-actin expression as a house keeping gene. ZAP KO cells were generated using the CRISPR-Cas9 system from Sant Cruz (sc-407495-KO-2). For western blot analysis cells were lysed in RIPA Lysis Buffer (Thermo Fisher), and subjected to SDS-PAGE on a Mini-Protein 4-12% gradiant  SDS polyacrylamide gel and immunoblotting. Nitrocellulose membranes were probed with primary antibody against ZAP (Abcam, ab154690; 1:1000 dilution) and β-actin (Abcam ab9484) conjugated with HRP. Gel was visualized on a ChemiDoc MP Imaging System (Bio-Rad).

B) Replication kinetics of CpG-high, CpG-low and A/WSN/33-WT viruses in A549 WT and A549 ZAP-KO cells inoculated at an MOI of 0.01. Supernatants were harvested every 12 hours for 3 days post inoculation and Titers (TCID50/mL) were determined in MDCK cells. A representative of three experiments in three independent KO clones is shown.

C) Area under the curve (AUC) values were compared between both cell types for each virus. Statistical significance was determined by one-way ANOVA with Tukey’s multiple-comparison test, where ns indicates non-significance and ******p ≤ 0.001.**
